# Evolving origin-of-transfer sequences on staphylococcal conjugative and mobilizable plasmids—who’s mimicking whom?

**DOI:** 10.1093/nar/gkab303

**Published:** 2021-05-03

**Authors:** Karina Yui Eto, Stephen M Kwong, Patrick T LaBreck, Jade E Crow, Daouda A K Traore, Nipuna Parahitiyawa, Heather M Fairhurst, D Scott Merrell, Neville Firth, Charles S Bond, Joshua P Ramsay

**Affiliations:** School of Molecular Sciences, University of Western Australia, Perth, WA 6009, Australia; Curtin Medical School, Curtin University, Perth, WA 6102, Australia; Curtin Health Innovation Research Institute, Curtin University, Perth, WA 6102, Australia; School of Life and Environmental Sciences, University of Sydney, Sydney, NSW 2006, Australia; Department of Microbiology and Immunology, Uniformed Services University of the Health Sciences, Bethesda, Maryland, MD 20814, USA; Curtin Medical School, Curtin University, Perth, WA 6102, Australia; Curtin Health Innovation Research Institute, Curtin University, Perth, WA 6102, Australia; Department of Biochemistry and Molecular Biology, Infection and Immunity Program, Monash Biomedicine Discovery Institute, Monash University, Clayton, VIC 3800, Australia; Faculty of Natural Sciences, School of Life Sciences, Keele University, Staffordshire ST5 5BG, UK; Life Sciences Group, Institut Laue Langevin, Grenoble 38000, France; Faculté des Sciences et Techniques, Université des Sciences, des Techniques et des Technologies de Bamako (USTTB), Bamako BP E423, Mali; Curtin Medical School, Curtin University, Perth, WA 6102, Australia; Curtin Medical School, Curtin University, Perth, WA 6102, Australia; Department of Microbiology and Immunology, Uniformed Services University of the Health Sciences, Bethesda, Maryland, MD 20814, USA; School of Life and Environmental Sciences, University of Sydney, Sydney, NSW 2006, Australia; School of Molecular Sciences, University of Western Australia, Perth, WA 6009, Australia; Curtin Health Innovation Research Institute, Curtin University, Perth, WA 6102, Australia; Curtin Medical School, Curtin University, Perth, WA 6102, Australia; School of Molecular Sciences, University of Western Australia, Perth, WA 6009, Australia

## Abstract

In *Staphylococcus aureus*, most multiresistance plasmids lack conjugation or mobilization genes for horizontal transfer. However, most are mobilizable due to carriage of origin-of-transfer (*oriT*) sequences mimicking those of conjugative plasmids related to pWBG749. pWBG749-family plasmids have diverged to carry five distinct *oriT* subtypes and non-conjugative plasmids have been identified that contain mimics of each. The relaxasome accessory factor SmpO, encoded by each conjugative plasmid, determines specificity for its cognate *oriT*. Here we characterized the binding of SmpO proteins to each *oriT*. SmpO proteins predominantly formed tetramers in solution and bound 5′-GNNNNC-3′ sites within each *oriT*. Four of the five SmpO proteins specifically bound their cognate *oriT*. An F7K substitution in pWBG749 SmpO switched *oriT*-binding specificity *in vitro*. *In vivo*, the F7K substitution reduced but did not abolish self-transfer of pWBG749. Notably, the substitution broadened the *oriT* subtypes that were mobilized. Thus, this substitution represents a potential evolutionary intermediate with promiscuous DNA-binding specificity that could facilitate a switch between *oriT* specificities. Phylogenetic analysis suggests pWBG749-family plasmids have switched *oriT* specificity more than once during evolution. We hypothesize the convergent evolution of *oriT* specificity in distinct branches of the pWBG749-family phylogeny reflects indirect selection pressure to mobilize plasmids carrying non-cognate *oriT*-mimics.

## INTRODUCTION

The majority of horizontally acquired antimicrobial-resistance genes in *Staphylococcus aureus* are located on circular DNA plasmids. An estimated ∼90% of *S. aureus* isolates possess at least one plasmid and ∼79% carry large (>20 kb) plasmids frequently harbouring collections of resistance and virulence genes (1). Evidence for the movement of plasmids between distinct lineages of *S. aureus* and their introduction from other species and genera is abundant (2). However, the majority of *S. aureus* plasmids lack conjugation genes and therefore must depend on self-transmissible mobile elements such as bacteriophage or conjugative plasmids for their horizontal transfer (1,3).

The pWBG749 family of plasmids are a recently characterized family of conjugative staphylococcal plasmids that are distinct from the well characterized pSK41/pGO1 family. pWBG749-family plasmids have been identified carrying genes for resistance to methicillin, vancomycin, penicillin, gentamicin, trimethoprim, mupirocin, cadmium and chlorhexidine (4–11). As well as directly disseminating antimicrobial resistance, pWBG749-family plasmids can mobilize numerous non-conjugative multiresistance plasmids (7,8). In most well-understood mechanisms of conjugative mobilization, a mobilizable plasmid carries both a DNA relaxase gene and an origin-of-transfer (*oriT*) sequence. The expressed relaxase protein recognizes and nicks the mobilizable plasmid *oriT* and recruits ssDNA to the conjugative-plasmid-encoded type IV secretion system for transfer. In contrast, documented plasmids mobilized by pWBG749, lack their own relaxase gene and instead carry sequence mimics of the pWBG749 origin-of-transfer (*oriT*). The pWBG749-encoded relaxase therefore acts *in trans* on the mobilizable-plasmid’s *oriT* mimic. An estimated 50% of sequenced non-conjugative *S. aureus* plasmids carry one or more pWBG749-like *oriT* sequences (7). *oriT* mimic-carrying plasmids have been subsequently identified in *Escherichia coli* and *Acinetobacter baumannii* (12–14), with ∼20% sequenced *A. baumannii* plasmids carrying *oriT* sequences resembling those on conjugative plasmids (15). A search for *oriT* sequences in 4602 plasmids spanning 22 distinct phyla revealed that while only 29% carried recognizable relaxase genes, 69% carried at least one *oriT* sequence (16). These observations suggest the largely unexplored ‘relaxase *in trans*’ mechanism of plasmid mobilization is an underappreciated route for horizontal gene transfer in both gram-positive and gram-negative bacteria (1,7).

**Figure 1.**
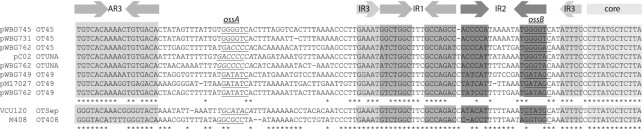
pWBG749-family *oriT* sequences examined in this work. Alignments of pWBG749-family *oriT* sequences examined in this work are shown; inverted repeats are shaded. The pWBG749-family *oriT* sequences include 1–3 copies of the AR repeat sequence (AR1-AR3); however, only the AR3 copy is essential for conjugative mobilization and AR1-AR2 are not shown here. The OTSep and OT408 sequences are aligned separately as they contain distinct AR sequences. Asterisks under alignments indicate positions with 100% nucleotide conservation. The IR2 sequences vary between *oriT* subtypes. Each of the *oriT* sequences shown are located on conjugative plasmids, except for those of pWBG762; pWBG762 carries mimics of OT49, OTUNa and OT45 (4,17). The 5′-GNNNNC-3′motifs central to each of the *oriT* specificity sequences *ossA* and *ossB* discovered in this work are underlined. The *ossA* sites are shown in italics if they are on the complementary strand relative to the *ossB* site.

The *oriT* sequences of pWBG749-family plasmids have diverged into at least five distinct subtypes based on nucleotide identity, previously named OT49, OT45, OTUNa, OT408 and OTSep (7) (Figure 1). Each *oriT* subtype contains an identical core sequence targeted by the putative relaxase SmpP and a conserved arrangement of DNA-repeat sequences. The repeat sequences IR1 and IR3 are largely conserved in sequence between subtypes, but the IR2 repeat sequences have diverged. pWBG749 carries an OT49 *oriT* and can mobilize plasmids carrying OT49 sequences, but not those carrying OT45 or OTUNa. Conversely, conjugative plasmids pC02 and pWBG731 carry OTUNa and OT45 *oriT* subtypes, respectively, and both can mobilize plasmids carrying either OTUNa or OT45 *oriT*, but not OT49. While the pC02 and pWBG731 plasmids share the same *oriT* specificities, their conjugation-gene sequences are more divergent in comparison to pWBG731 and pWBG749 (17), suggesting that *oriT* specificity of each plasmid has evolved somewhat independently of rest of the conjugation-gene cluster. These same five *oriT* subtypes are also present as *oriT* mimics on non-conjugative plasmids and enable mobilization by pWBG749 plasmids with a matching *oriT* sequence subtype specificity (4,7,17). Some plasmids have captured 2 or 3 *oriT* mimic sequences and most often they are of different subtypes. The observation that non-conjugative plasmids have captured and retained these distinct *oriT* variants suggests these plasmids have benefited from the horizontal mobilization facilitated by various pWBG749-family members carrying these distinct *oriT*s during their evolutionary history.

A conjugative plasmid’s ability to discriminate between its cognate *oriT* and other DNA sequences is presumably advantageous for both a conjugative plasmid and the bacterial host (18). Relaxase-*oriT* recognition often involves additional accessory DNA-binding proteins or ‘relaxosome accessory factors’ (RAFs), which can be essential for efficient *oriT* recognition and nicking by the relaxase. Characterized RAFs frequently contain a ribbon-helix-helix (RHH) DNA-binding domain within their N-termini, including TraY_F_, TraM_F_, TrwA_R388_, NikA_RP64_, TraJ_RP64_, MobC_RSF1010_ and MbeC_ColE1_ in gram-negative bacteria, and PcfF_pCF10,_ Aux1_pLS20_, Ausx2_pLS20_ and SmpO_pWBG749_ in gram-positive bacteria (7,19–27). RAFs generally bind sequence(s) within or 5′ of the *oriT nic* site. RHH domains contain an antiparallel β-sheet formed from the N-termini of the two RHH monomers, which recognizes specific DNA base-pairs in the major groove. RAFs may also interact with the relaxase and/or the type-IV secretion system coupling protein (28).

The pWBG749-family SmpO proteins are RHH-containing RAFs encoded between the *oriT* site and downstream *smpP* relaxase gene on each pWBG749-family plasmid. While pWBG749 does not normally mobilize plasmids carrying the OT45 *oriT* it can efficiently mobilize an OT45 *oriT*-carrying plasmid if the plasmid additionally carries the *smpO*_45_ gene (7). This indicates variations in the *smpO* genes are likely responsible for differences in mobilization specificity between various pWBG749-family plasmids. In this work, we identified the DNA-binding sites for five distinct SmpO proteins using surface plasmon resonance (SPR) and found SmpO proteins specifically bind two sites within each *oriT*. Amino acid substitutions in SmpO_49_ and SmpO_45_ revealed a single change switched *oriT* specificity *in vitro* and *in vivo* and phylogenetic comparisons of conjugation-gene sequences suggested similar changes have occurred in divergent members of the pWBG749 family of conjugative plasmids.

## MATERIALS AND METHODS

### Conjugation and mobilization

Conjugation experiments were carried out as previously described (7).

### Strain and plasmid construction

Construction and sequencing of pLI50 plasmid constructs was carried out as previously described (7,17) Details of strains, plasmids and their construction can be found in Supplementary Table S1 and oligonucleotides used for cloning can be found in Supplementary Table S2. For construction of pWBG749e-F7K, the *smpO*_49-F7K_ allele was introduced by allelic exchange using pIMAY-Z (29). Oligonucleotides #68–71 (Supplementary Table S1) were used to amplify two overlapping fragments (1–2 and 3–4) from pWBG749e. The two fragments and linearized pIMAY-Z were then assembled using Gibson Assembly (NEB). The desired pIMAY-Z construct was validated by DNA sequencing, then introduced into *S. aureus* WBG4515 harbouring pWBG749e. Blue colonies on NYE agar plates containing erythromycin (5 μg/ml), chloramphenicol (10 μg/ml) and X-gal (40 μg/ml) were switched between permissive and non-permissive temperatures for pIMAY-Z replication to induce integration, excision and then plasmid loss as described previously (29). The pWBG749e-F7K plasmid was screened by PCR and confirmed by Sanger sequencing.

### Protein purification

SmpO_49_ and SmpO_45_ coding sequences were amplified by PCR respectively from pWGB749 and pWBG745. SmpO_49-F7K_ and SmpO_45-K7F_ coding sequences were amplified from pLIOT5S9M-F7K and pLIOT9S5M-K7F. Coding sequences for other SmpO variants SmpO_UNa_, SmpO_Sep_ and SmpO_408_ were codon optimized using JCaT (http://www.jcat.de) (30) and synthesized (IDT). PCR products and synthesized DNA fragments were digested and cloned into NcoI/BamHI or XbaI/BamHI sites of pETM-11. All constructs were introduced to *E. coli* BL21(DE3)pLysS by electroporation. Transformed cells were grown in 1 L of LB supplemented with chloramphenicol (Cm) at 100 μg/ml and kanamycin (Km) at 50 μg/ml and incubated at 25°C and 200 rpm shaking. At an optical density at 600 nm of approximately 0.6–0.8, cells were induced with isopropyl-β-D-1-thiogalactopyranoside (IPTG) at 0.5 mM final concentration for 16 h at 18°C and 180 rpm shaking. The cells were harvested by centrifugation at 8000 *g* for 20 min at 4°C; 1 L culture cell pellets were gently washed in nickel binding buffer [50 mM NaH_2_PO_4_, pH 7.5, 1 M NaCl, 10% (v/v) glycerol, 25 mM imidazole] and centrifuged again at 8000 *g* for 20 min at 4°C; cell pellets were immediately used or stored at -80°C. The cell pellets were resuspended in 50 ml of nickel-affinity binding buffer supplemented with 2 μg/ml of DNAse I nuclease and 300 μg/ml of lysozyme. Lysis was carried out with Emulsiflex C5 high-pressure homogeniser (Avestin), and the lysate was clarified by centrifugation (24 000 *g* for 45 min at 4°C). The clarified lysate was filtered (0.22 μm) prior to application onto 5 ml NiCl_2_-charged HisTrap HP column (GE Healthcare). The cleavable hexahistidine-tagged SmpO protein (6H-SmpO) was eluted using an imidazole gradient (25–500 mM) over eight column-volumes. Eluted 6H-SmpO protein was diluted to 2 mg/ml with TEV protease digestion buffer [50 mM Tris-HCl pH 7.5, 250 mM NaCl, 1 mM EDTA, 5 % (v/v) glycerol, 1 mM DTT], uncleavable hexahistidine-tagged TEV protease (produced in-house) was added at protease:SmpO ratio of 1:10 (w/w), then dialysed into TEV protease digestion buffer for 16 h at ambient temperature with gentle mixing. Post-digestion, dialysed SmpO TEV protease digestion reaction was centrifuged (24 000 *g* for 10 min at 4°C), 0.22 μm filtered and re-applied to the HisTrap column. The flowthrough containing the tag-cleaved SmpO protein was further purified using a HiLoad 16/60 Superdex 75 column (GE Healthcare) preequilibrated in SEC buffer [50 mM Tris-HCl, pH 7.5, 1 M NaCl, 1 mM EDTA, 5% v/v glycerol]. The concentration was determined from absorption at 280 nm using absorption coefficients and theoretical molecular mass values calculated from sequence using *ExPasy Protparam* (31). Purified SmpO proteins were used in following experiments or flash-frozen with liquid nitrogen for long-term storage at -80°C. Chromatography purification steps were performed using AKTA Start and/or AKTA purifier FPLC system (GE Healthcare) at 4°C and absorbance traces at 280 nm only (AKTA Start), or 280, 260 and 230 nm (AKTA Purifier) were constantly monitored.

### SEC-MALS

All SEC-MALS experiments were carried out using Superdex Increase 10/300 GL column (GE Healthcare) attached to Viskotek GPCmax VE 2001 solvent/sample module (Malvern) coupled to Viskotec 305 TDA detector array (Malvern) at room temperature. In summary, 200 μl of purified SmpO protein samples at 2–3 mg/ml in MALS buffer [10 mM HEPES, pH 7.5, 250 mM NaCl, 3 mM EDTA, 2.5 % (v/v) glycerol, 0.05% (v/v) Tween 20] and bovine serum albumin (BSA) (Sigma) samples of approximately 1 mg/ml in MALS buffer were applied to the size-exclusion column pre-equilibrated with MALS buffer at flow rate of 0.3 ml/min, monitoring the refractive index, UV absorbance and left and right-angle light scattering. OmniSEC 5.10 Bio software (Malvern) was used to analyse SEC profile and to calculate molecular weight averages and dispersity using calibration settings derived from five BSA samples, then results were averaged.

### Electrophoretic mobility shift assays (EMSAs)

The *oriT*_45_ and *oriT*_49_ regions were PCR amplified from pWBG745 and pWBG749e using primers carrying 3′ adapter sequences (Supplementary Table S3). The PCR products were used as competitive unlabelled DNA in the EMSAs and amplified using IRDye800-labelled primers targeting the tag-sequences (Supplementary Table S3). Binding reactions were set up as per Li-Cor^®^ EMSA using IRDye® oligonlucleotides protocol, except that reaction mixtures contained 0.5 μg/μl sheared Herring sperm DNA, IRDye800-labelled DNA at 5 nM final concentration. SmpO_45_, SmpO_49_ and SmpO_49-F7K_ were diluted into 1× binding buffer containing 2.5% glycerol and 0.05% Tween 20, then added to the binding reactions at various concentrations. Native acrylamide/agarose hybrid gel electrophoresis was carried out in pre-run gels containing 3% 19:1 acrylamide:bis-acrylamide, 0.5% agarose, 0.5× TBE buffer (1× TBE = 89 mM Tris, 89 mM boric acid, 2 mM EDTA), 2.5% glycerol, run in 0.5× TBE buffer.

### SPR-based DNA footprinting assays

All SPR experiments were carried out using a Biacore T200 (GE Healthcare), Biacore SA sensor chip (GE Healthcare) and applying the Re-usable DNA Capture Technique (ReDCaT) (32). The oligonucleotide arrays for the *oriT* sequences from pWBG749e, pWBG745, pC02 and *S. aureus* strain M0408 (GI:477787193) were designed using the Perl script *poop.pl* (32), and *ossA*_Sep_ and *ossB*_Sep_ sequences were originated from *S. aureus* strain VCU120 (GI:41866860). The oligonucleotides used on SPR assays are listed in Supplementary Table S3. A DNA-binding affinity protocol was derived from the method of Stevenson *et al.* (32). In each cycle of screening assays, unless stated, cycle steps were run at 30 μl/min; test DNA was capture on Fc2, and control ReDCaT complementary linker was capture on Fc1 both at 1 μM and 10 μl/min; subsequently SmpO protein at 1 μM was injected onto Fc1 and Fc2 for 60 s contact time and 60 s dissociation time, followed by a wash with regeneration buffer [50 mM NaOH, 1 M NaCl] and 90 s stabilization with SPR buffer [10 mM HEPES, 150–300 mM NaCl, 3 mM EDTA, 0.05% (v/v) Tween 20]. To maintain SmpO protein stability in solution and optimise DNA-binding in SPR, SmpO_45_, SmpO_408_ and SmpO_UNa_ SPR screening assays were carried out in SPR buffer containing 300 mM NaCl, while SmpO_49_ SPR screening assays were carried out in SPR buffer with 150 mM NaCl. Assays presented in Supplementary Table S4 where all five SmpO proteins were tested together were carried out in SPR buffer containing 250 mM NaCl. Complete SPR data are also presented in Supporting Dataset S1.

## RESULTS

### SmpO proteins bind two *oriT*-specificity-sites *ossA* and *ossB*

Five distinct pWBG749-family SmpO proteins SmpO_49_ (Genbank accession ACZ58780 from plasmid pWBG749), SmpO_45_ (ACZ58661 from pWBG745, identical to SmpO of pWBG731; AXQ85809), SmpO_UNa_ (AKR47467 from plasmid pC02), SmpO_408_ (ENK02073 from *S. aureus* MO408) and SmpO_Sep_ (EHR84364 from *S. epidermidis* VCU120) were purified using nickel-affinity chromatography and size-exclusion chromatography (SEC) (see ‘Materials and Methods’ section). SEC coupled with multi-angle light scattering (SEC-MALS) analysis revealed SmpO_49_, SmpO_45_, SmpO_408_ and SmpO_Sep_ each existed predominantly as tetramers (84–96.6%) in solution, with smaller proportions as hexamers (3.4–16%) (Supplementary Table S5). We were unable to purify enough SmpO_UNa_ for SEC-MALS.

**Figure 2.**
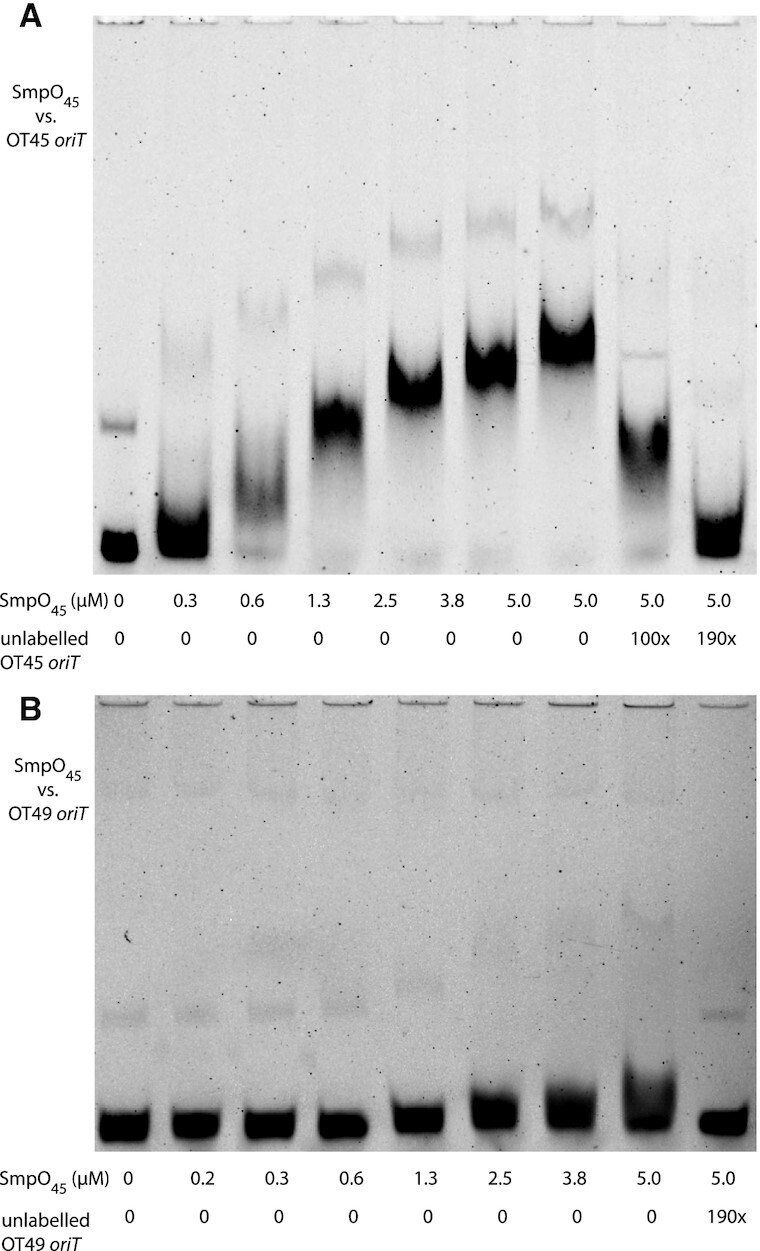
EMSA of SmpO_45_ with *oriT* regions amplified from OT45 and OT49. Purified SmpO_45_ was used in EMSA assays with a 5 nM of a 134-bp dual-IRDye800-labelled DNA region encompassing AR3-IR3 for *oriT* sequences OT45 and OT49 (Figure 1). (**A**) EMSA with SmpO_45_ and labelled OT45. The final two lanes include unlabelled competitor OT45 DNA. (**B**) EMSA with SmpO_45_ and labelled OT49. The final lane includes unlabelled competitor OT49 DNA. Secondary labelled DNA fragments with an apparent higher molecular weight were visible for both labelled OT45 and OT49 and were shifted in a similar manner to the main PCR products.

We first examined binding of SmpO_45_ to the pWBG745 OT45 *oriT* (*oriT*_45_) using EMSAs. IRDye800-labelled oligonucleotides were used to PCR-amplify a 134-bp DNA fragment from the *oriT*_45_ sequence encompassing repeats AR3-IR3 (Figure 1). Purified SmpO_45_ induced a series of DNA-migration shifts in the presence of increasing concentrations of SmpO_45_ (Figure 2A). Addition of excess unlabelled *oriT*_45_ DNA outcompeted labelled DNA and reversed the shifts. EMSAs using SmpO_45_ with IRDye800-labelled *oriT*_49_ DNA produced only a minor shift at high SmpO_45_ concentrations, confirming SmpO_45_ exhibited higher specificity for its cognate *oriT*_45_ sequence than the non-cognate *oriT*_49_ (Figure 2B). The PCR products for both *oriT*_45_ and *oriT*_49_ contained a minor secondary species exhibiting slower gel migration that also shifted in EMSAs with increasing SmpO_45_ concentrations. Gel purification did not remove this DNA species nor did denaturing and reannealing the DNA. This secondary product also increased with PCR-product age, suggesting some alternate secondary DNA structure formed during storage. The IR1–IR3 repeats are structurally conserved in *oriT* regions of diverse gram-positive conjugative plasmids and potentially form a ssDNA branched hairpin structure, so it is possible that these secondary structures could be the cause of this secondary band in EMSAs (7). In summary, the complex series of DNA-shifts for the *oriT*_45_ region with SmpO_45_ suggested there may be multiple binding sites for SmpO_45_ in the region, multiple DNA isoforms within complexes and/or that SmpO_45_ formed higher-order oligomers or soluble aggregates with DNA at higher concentrations.

**Figure 3.**
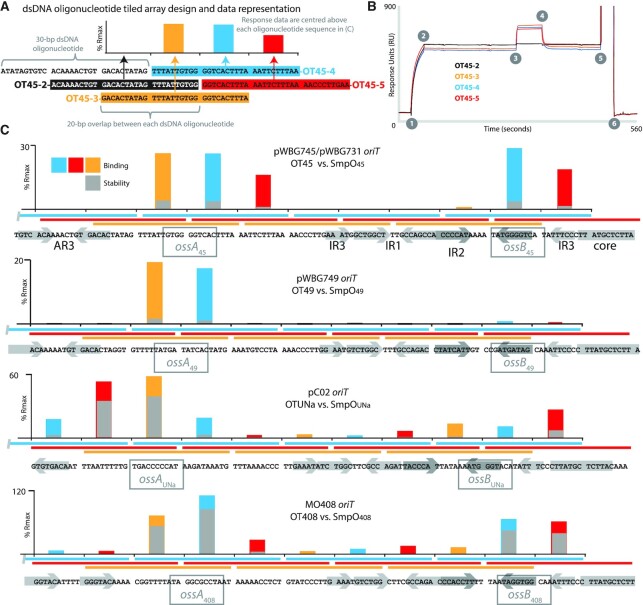
Surface plasmon resonance DNA-footprinting of SmpO and *oriT* regions. (**A**) Diagram outlining the design of dsDNA oligonucleotide arrays for each of the *oriT* regions using dsDNA oligonucleotides OT45–2, OT45–3, OT45–4 and OT45–5 as examples. SPR *R*_max_ values are indicated in the graph above, with each bar corresponding to the oligonucleotide centred at that position and highlighted in the same colour. (**B**) Example SPR response data corresponding to oligonucleotides as labelled in (A). Numbers on the graph indicate different steps in the SPR assay process. (1) DNA capture, (2) end of DNA capture flowcell wash, (3) addition of 1 μM purified SmpO protein, (4) protein addition ceases, (5) flowcell regeneration step starts, removing dsDNA oligonucleotide and (6) regeneration step ceases and flowcell is washed with SPR buffer prior to the next cycle with the next oligonucleotide. Responses are measured between steps 3 and 4 from the baseline established between steps 2 and 3. (**C**) SPR binding and stability responses for 1 μM of each purified SmpO protein to *oriT* oligonucleotide arrays for OT45, OT49, OTUNa and OT408 (from top to bottom). The sequence of each *oriT* array examined is shown at the bottom of each graph and AR3, IR1-IR3 and the core regions are shaded. Between each graph and *oriT* sequence are coloured bars representing the dsDNA oligonucleotides tested in each SPR array, corresponding to the sequence directly below and the similarly coloured bars in the graph above each set of tiles. SPR responses (coloured bars) are expressed as a percentage of *R*_max_, the theoretical maximum binding response expected assuming a tetrameric SmpO protein binds to a single dsDNA oligonucleotide, and are calculated from the molecular weights of the oligonucleotides and SmpO tetramers. Stability measurements (grey bars) are the measured *R*_max_ response 10 s after step 4 indicated in part (B). Complete SPR data are presented in Supporting Dataset S1.

To identify sequences recognized by each SmpO protein, DNA-binding assays were carried out by SPR using ‘Reusable DNA Capture Technology (ReDCaT)’ (32,33). Briefly, a biotinylated ssDNA oligonucleotide was permanently immobilized to a streptavidin-coated SPR chip (the ReDCaT chip), and dsDNA oligonucleotides with a 3′ overhang complementary to the biotinylated oligonucleotide were successively bound to and released from the ReDCat chip. The SPR response for each oligonucleotide, corresponding to a change in mass on the chip's surface, was used as a baseline from which SPR responses were measured following addition of purified SmpO protein (see ‘Materials and Methods’ section and Figure 3). Tiled arrays of 30-bp dsDNA oligonucleotides spanning the OT45, OT49, OTUNa and OT408 *oriT* sequences were synthesized (Figure 3A). For SmpO_45_ (Figure 3), SPR responses corresponding to ∼30% of the theoretical maximum response (*R*_max_) were detected with oligonucleotides spanning two distinct regions within the OT45 *oriT*, named here ‘*oriT* specificity sequence A’ (*ossA*_45_) and *ossB*_45_. *ossB*_45_ was located within the right-hand arm of the IR2 repeat sequence proximal to the core region, confirming our previous speculations that the more divergent IR2 repeats within each *oriT* were involved in mobilization specificity (7,17). The *ossA*_45_-binding site was positioned ∼50-bp upstream of IR2, between AR3 and IR3. Both *ossA*_45_ and *ossB*_45_ contained the sequence 5′-TGGGGTCA-3′. Reinspection of each of the other four *oriT* subtypes revealed they too carried sequences in a similar position that resembled the arm sequences of the IR2 region. The putative *ossA* sites for SmpO_Sep_ and SmpO_UNa_ were present on the opposite DNA strand relative to their respective *ossB* site sequences (Figures 1 and 3). SPR footprinting with purified SmpO_49_, SmpO_UNa_ and SmpO_408_ proteins and their corresponding oligonucleotide arrays confirmed they all also bound their respective *ossA* regions; however, SmpO_49_, SmpO_UNa_ and SmpO_408_ each exhibited weaker responses with their respective *ossB* sites.

**Figure 4.**
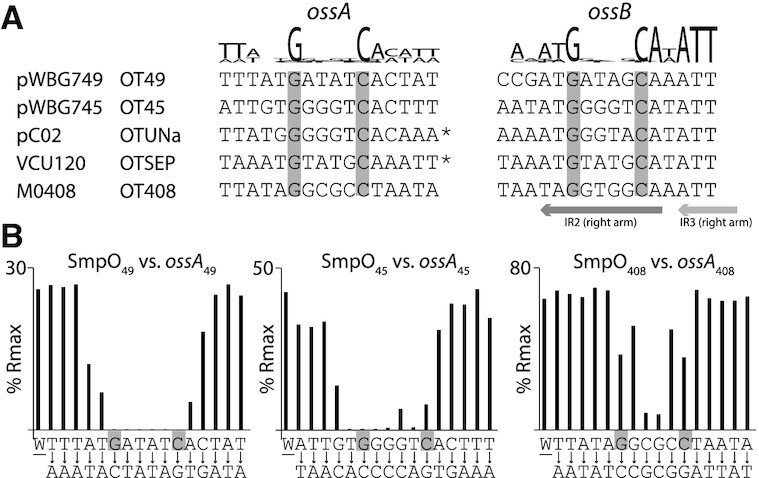
Single-nucleotide mutagenesis of OT49, OT45 and OT408 *ossA* sites. (**A**) An alignment of *ossA* and *ossB* sequences for each of the five *oriT* sequences examined in this work. The conserved G and C nucleotides centred about each *oss* site are highlighted in grey and a sequence logo indicating nucleotide conservation is shown above each alignment. The positions of the right-arms of the IR2 and IR3 repeats are indicated below as arrows. (**B**) Single-nucleotide mutagenesis of *ossA* sites and SPR responses with SmpO proteins. Sixteen-base-pair sequences containing *ossA*_49,_*ossA*_45_ and *ossA*_408_ were synthesized and assayed by SPR with 1 μM of each cognate purified SmpO protein as indicated. W indicates the SPR response for the wild-type sequence, while each of the other sequence letters represent a dsDNA oligonucleotide containing a single base-pair substitution to the complementary base-pair at that position as indicated below each base. Complete SPR data are presented in Supporting Dataset S1.

To refine the DNA sequences critical for binding by each SmpO protein, we first aligned all the *ossA* and *ossB* sites to identify any common features of the *oss* sites. This revealed a conserved 5′-GNNNNC-3′ motif present within all *ossA* and *ossB* sites, which was often centred within or around a distinct region of dyad symmetry in each sequence (Figure 4A). We then designed 16-bp dsDNA oligonucleotides containing each GNNNNC motif for *ossA*_49_, *ossA*_45_ and *ossA*_408_. For each of these 16-bp *ossA* regions, we designed a further 16 dsDNA oligonucleotides each containing a single-base-pair substitution to the complementary base-pair for each of the 16 nucleotide positions (Figure 4B). SPR assays using these oligonucleotides with their corresponding SmpO proteins demonstrated that mutations within each GNNNNC motif were most detrimental to SmpO binding. Finally, to examine the specificity of SmpO binding for cognate *ossA* and *ossB* sites, 16-bp dsDNA oligonucleotides for each of *ossA* and *ossB* site, including predicted GNNNNC sites for the OTSep *ossA* and *ossB* sites (Figure 4A), were used in SPR experiments with each of the five purified SmpO proteins. To enable an unbiased comparison between all the proteins in a single SPR run, SPR buffer containing an intermediate but suboptimal NaCl concentration (250 mM) was used, which unfortunately substantially reduced several of the SPR responses. However, despite these limitations, each SmpO protein exhibited the strongest SPR responses for its cognate *ossA* and *ossB* sequences (Supplementary Table S4). SmpO_UNa_ also exhibited a binding response with *ossA*_45_, consistent with the sequence similarity between the *ossA*_Una_ and *ossA*_45_ sites and the ability of pC02 to mobilize plasmids carrying OTUNa or OT45 *oriT* subtypes (4,17).

### Incongruence between conjugative-plasmid phylogeny and *oriT* specificity

**Figure 5.**
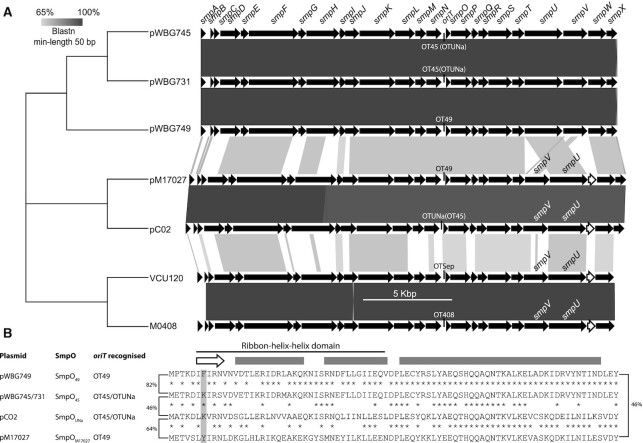
Incongruence between conjugation-gene clusters and their *oriT* specificity. (**A**) The pWBG749-family conjugation-gene clusters (*smpA-smpX*) of pWBG749 (GQ900391), pWBG745(GQ900389), pWBG731 (MH587574), pM17027 (JGJU01000023.1), pC02 (CP012121), *S. epidermidis* VCU120 (AHLC01000011) and *S. aureus* MO408 (AIWO01000029) were aligned for tree construction using MEGA X (34). Black arrows indicate conserved conjugation genes *smpA-X* and the white arrows indicate a unique gene present in the pM17027, pC02, VCU120 and M0408 clusters, typified by the pC02 locus tag ACO02_2800. A Maximum Likelihood tree based on the Tamura-Nei model (35) was constructed with 1000 replicates and 100% of trees supported all nodes (36). The conjugation-gene cluster alignment was constructed using Easyfig and BLASTN (37). (**B**) Sequence alignments of the SmpO proteins from pWBG749 versus pWBG745/pWBG731, pWBG745/pWBG731 versus pC02 and pC02 versus pM17027. Asterisks indicate identical residues conserved between each two aligned sequences. PSI-PRED secondary-structure prediction is shown above (the arrow indicates the β-sheet region and bars represent helices) and residue 7 of the SmpO proteins is shaded. Brackets either side of the sequences indicate % amino acid identity between sequences.

As noted here and previously (4,17), there are clear incongruences between the nucleotide sequence similarities of pWBG749-family conjugation-gene clusters and the *oriT* subtypes they carry. Alignment of the conjugation-gene cluster sequences carrying each of the five *oriT* subtypes (Figure 5A) (34–37) clustered the pWBG731/pWBG745 conjugation genes with those of pWBG749, despite these plasmids carrying distinct *oriT* recognized by SmpO proteins with distinct specificities. Conversely, the pC02 conjugation-gene cluster has diverged from pWBG731/pWBG745 both in nucleotide sequence similarity and gene order, yet these conjugative plasmids mobilize the same *oriT* sequence subtypes (OT45 and OTUNa). To search for other such discrepancies, we carried out BLASTN searches against whole-genome shotgun sequences using the *smpN-oriT-smpO* region from pC02 and looked for sequence divergence in the *oriT* region. A 39-kb contig from *S. aureus* M17027 (JGJU01000023.1) carrying a complete *smpA-X* cluster was identified. Nucleotide alignments of pC02 and pM17027 revealed the *smpA-smpN* and *smpP-smpU* regions share 96–99% nucleotide identity, while the *oriT* sequence (aligned from AR3-core, Figure 1) and *smpO* genes share only 67% and 68% nucleotide identity, respectively. The pM17027 *oriT* sequence contained the *ossA*_49_ and *ossB*_49_ sequences 5′-GATATCA-3′ and 5′-GATAGCA-3′, identical to those on pWBG749, not the *ossA*_Una_ and *ossB*_UNa_ sequences present on pC02. Alignment of the pM17027 and pWBG749 *oriT*s revealed they share 78% nucleotide identity, but the *smpO* sequences from these same plasmids share only 59% identity.

Next, we investigated whether the *smpO* alleles carried by pWBG749 and pM17027 could enable mobilization via the OT49 *oriT* by pWBG731 and pC02 (Table 1). Vectors containing the pWBG749 *oriT* positioned upstream of cloned *smpO*_45_, *smpO*_49_ or synthesized *smpO*_M17027_, were introduced into *S. aureus* RN4220, after which pWBG731 or pC02 were introduced into each strain to produce conjugative donors. As expected, pWBG731 and pC02 did not mobilize a plasmid carrying the OT49 *oriT* alone or a plasmid carrying the OT49 *oriT* with the *smpO*_45_ gene. However, both pWBG731 and pC02 mobilized plasmids carrying the OT49 *oriT* when the vector also carried *smpO*_49_ or *smpO*_M17027_. These results confirmed that the newly identified *smpO*_M17027_ enabled recognition of the OT49 *oriT* and that the two distantly-related plasmids pWBG731 and pC02 both mobilized plasmids carrying the non-cognate OT49 *oriT* if provided a compatible *smpO* gene.

**Table 1.** Mobilization of OT49-*oriT*-carrying plasmids by pWBG731 and pC02

Construct name	*oriT*	SmpO	pWBG731 conjugation^a^	pWBG731 mobilization	pC02 conjugation	pC02 mobilization
pLI50	None	None	2.8 × 10^−4^ (± 4.2 × 10^−4^)	<1.0 × 10^−9^	1.5 × 10^−4^ (± 5.5 × 10^−5^)	<1.0 × 10^−9^
pKY5T	OT45	None	1.2 × 10^−4^ (± 1.7 × 10^−4^)	1.4 x 10^−4^ (± 1.5 × 10^−4^)	5.9 × 10^−4^ (± 4.8 × 10^−4^)	1.6 × 10^−5^ (± 2.5 × 10^−5^)
pKY5TO	OT45	SmpO_45_	4.6 × 10^−5^ (± 1.0 × 10^−5^)	2.1 × 10^−5^ (± 1.4 × 10^−5^)	3.1 × 10^−4^ (± 2.6 × 10^−4^)	5.5 × 10^−6^ (± 5.3 × 10^−6^)
pLI749a	OT49	None	8.4 × 10^−5^ (± 7.1 × 10^−5^)	<1.0 × 10^−8^^c^	2.5 × 10^−4^ (± 3.2 × 10^−4^)	<1.0 × 10^−9^
pLIOT9S5-N-WT45	OT49	SmpO_45_	7.5 × 10^−5^ (± 7.8 × 10^−5^)	<1.0 × 10^−8^^c^	1.6 × 10^−3^ (± 2.3 × 10^−3^)	<1.0 × 10^−9^
pLIT9O99	OT49	SmpO_49_	2.0 × 10^−4^ (± 7.3 × 10^−5^)	1.9 × 10^−4^ (± 1.2 × 10^−4^)	2.0 × 10^−4^ (± 2.4 × 10^−4^)	2.2 × 10^−6^ (± 3.0 × 10^−6^)
pLIOT9S5-N-WT49	OT49	^b^SmpO_49_(1–17)::SmpO_45_(18–84)	1.1 × 10^−4^ (± 1.0 × 10^−4^)	5.7 × 10^−5^ (± 2.8 × 10^−5^)	3.1 × 10^−4^ (± 2.3 × 10^−4^)	1.6 × 10^−6^ (± 1.2 × 10^−6^)
pLIOT9S5M-K7F	OT49	SmpO_45-K7F_	6.1 × 10^−4^ (± 6.1 × 10^−4^)	4.2 × 10^−5^ (± 3.0 × 10^−5^)	3.4 × 10^−4^ (± 3.1 × 10^−4^)	5.8 × 10^−7^ (± 4.3 × 10^−7^)
pLIOT9S17027	OT49	SmpO_M17027_	3.2 × 10^−3^ (± 2.2 × 10^−4^)	2.4 × 10^−5^ (± 8.3 × 10^−6^)	6.9 × 10^−3^ (± 2.6 × 10^−3^)	4.3 × 10^−6^ (± 1.3 × 10^−6^)

^a^Per-donor transfer frequencies are the average of three independent experiments (±standard deviation).

^b^Numbers in brackets next to SmpO correspond to regions derived from SmpO_49_ or SmpO_45_ as indicated.

^c^Sporadic presence of 1 to 3 colonies from 100 µl of undiluted culture.

The amino acid sequence similarities between the SmpO proteins also appeared incongruent with the *oriT* sequences they mobilized (Figure 5B). SmpO_49_ and SmpO_45_ share 82% amino acid identity, yet they recognize distinct *oriT* sequences (OT49 and OT45/OTUNa, respectively). Conversely, SmpO_49_ shares only 46% amino acid identity with SmpO_M17027_ and yet they both enabled plasmid mobilization via the OT49 *oriT* (Table 1). SmpO_M17027_ instead appears more closely related in sequence to SmpO_UNa_ (64% identity Figure 5B), in line with relatedness of the other pM17027 and pC02 conjugation-gene sequences. These observations, taken together, suggested that members of the pWBG749-family of conjugative plasmids have switched *oriT* specificity between OT49 and OT45/OTUNa at least once in the pWBG749/pWBG745/pWBG731 and/or the pC02/pM17027 branches of the pWBG749 family (Figure 5A).

### A single amino acid substitution in SmpO switches mobilization specificity

**Figure 6.**
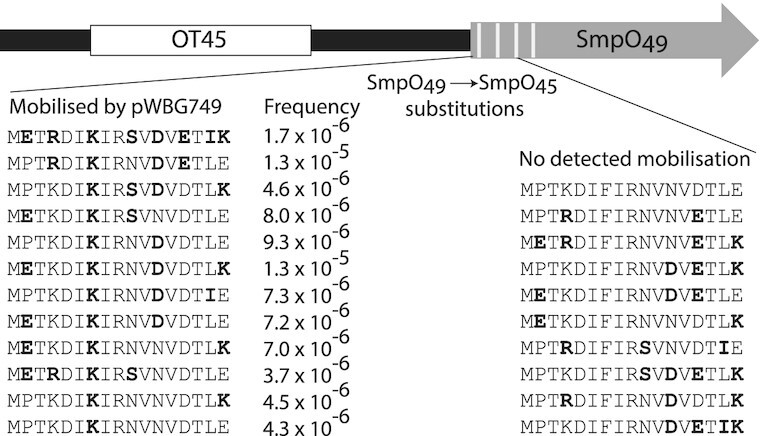
SmpO_49_ mutagenesis and pWBG749e-mediated mobilization via the OT45 *oriT*. The OT45 *oriT* sequence from pWBG745 was cloned upstream of the SmpO_49_ coding sequence. The first 17 amino acids of the SmpO_49_ coding sequence were semi-randomly mutated to match those present in SmpO_45_ as indicated in bold. Transfer frequency rates are given as exconjugants per donor and represent the average of three experiments. The complete dataset and construct names are presented in Supplementary Table S6.

Given that SmpO_49_ and SmpO_45_ share 86% amino-acid identity, we reasoned that *oriT* specificity differences between the two proteins involved a small number of amino acid residues. SmpO_49_ and SmpO_45_ differ by 8 of the first 17 amino acid residues, corresponding with the predicted β-sheet and first α-helix of the RHH domain, but only by 4 of the following 67 residues (Figure 5B). To determine if the first 17 residues of SmpO governed *oriT* specificity, we replaced the coding sequence for the first 17 amino acids of SmpO_49_ with that of SmpO_45_ and placed this gene downstream of the OT45 *oriT* (Figures 5B and 6). This plasmid (pLIOT5S9M-N-WT45) was mobilized by pWBG749e (8) with similar efficiency to a plasmid carrying the wild-type *smpO*_45_ gene (pKY5TO) (Figure 6 and Supplementary Table S6). Likewise, a construct carrying the OT49 *oriT* and a gene fusion encoding the first 17 amino acids of SmpO_49_ fused the last 67 residues of SmpO_45_ (pLIOT9S5-N-WT49) was mobilized by both pWBG731 and pC02 with similar efficiency to a plasmid carrying OT49 and the complete *smpO*_49_ gene (pLIT9O99) (Table 1). These experiments demonstrated the SmpO residues discriminating between the OT49 and OT45 *oriT* sequences are located within the first 17 amino acids of each protein.

We next constructed a range of mutant *smpO*_49_ alleles, each encoding 2–4 amino acid substitutions to match residues present in SmpO_45_ (Figure 6). Eleven out of twenty constructs were mobilized by pWBG749e and each of the mobilized plasmids carried a F7K substitution (Figure 6 and Supplementary Table S6). A construct encoding SmpO_49_ with an F7K substitution alone (SmpO_49-F7K_) was also mobilized as efficiently as a vector carrying wild-type *smpO_45_*. Similarly, we demonstrated that *smpO*_45_ containing an encoded K7F substitution (SmpO_45-K7F_) enabled mobilization of a plasmid carrying the OT49 *oriT* by pWBG731 and pC02 (Table 1). In summary, this single amino acid position in both SmpO_49_ and SmpO_45_ appeared responsible for the differences in specificity between OT49 and OT45 *oriT* subtypes.

To see if the *in vitro* DNA-binding specificity of the substituted SmpO proteins mirrored the observed changes in mobilization specificity, SmpO_49-F7K_ and SmpO_45-K7F_ were purified and tested for their ability to bind each of the *ossA* and *ossB* sequences. Both proteins formed tetramers and hexamers in similar proportions to their wild-type counterparts in SEC-MALS experiments (Supplementary Table S5), confirming changes did not alter the oligomerization state of the proteins. While we were unable to detect any DNA binding for SmpO_45-K7F_ in SPR experiments or EMSAs (not shown), the SmpO_49-F7K_ protein bound the non-cognate *ossA*_45_ and *ossB*_45_ sequences as strongly as SmpO_45_ (Supplementary Table S4). Moreover, while SmpO_49_ was unable to shift OT45 DNA in EMSA assays (Supplementary Figure S1A), SmpO_49-F7K_ produced a similar series of DNA shifts (Supplementary Figure S1B) to that of SmpO_45_ (Figure 2A). We did not detect any SmpO_49-F7K_ binding to the *ossA*_49_ and *ossB*_49_ sequences, confirming the gain in affinity of SmpO_49-F7K_ for the OT45 *ossA* and *ossB* sites was concomitant with a loss in affinity for the OT49 *ossA* and *ossB* sites. Therefore, these experiments confirmed (at least for SmpO_49_) that the changes in mobilization specificity of the F7K substitution were also reflected by DNA-binding specificity changes *in vitro*.

To investigate if the F7K change affected the ability of pWBG749 to recognize its own *oriT* in mobilization experiments we introduced the F7K change into *smpO*_49_ on pWBG749e, producing pWBG749e-F7K. Surprisingly, conjugation was only decreased 17-fold by this mutation (Table 2). Furthermore, when pWBG749e-F7K was tested in mobilization experiments with each of the cloned *oriT* mimics from pWBG762 (8), it mobilized all three plasmids (Table 2). The mobilization rate was highest for OT45, then OTUNa, and lowest for OT49, confirming that the *oriT* preference had indeed been reversed by the F7K change but that *in vivo* recognition of the OT49 *oriT* was not completely abolished.

**Table 2.** Mobilization of *oriT*-mimic-carrying plasmids by pWBG749e-F7K

	>	>	Transfer frequency*	
Construct name	Conjugative plasmid	Clone *oriT* mimic subtype	Conjugative plasmid	pLI50 clone
pLI50	pWBG749e	none	4.4 × 10^−3^ (4.4 × 10^−3^)	<1.0 × 10^−9^
pLI762–49	pWBG749e	OT49	4.3 × 10^−3^ (9.8 × 10^−4^)	2.1 × 10^−5^ (9.6 × 10^−6^)
pLI762–45	pWBG749e	OT45	6.2 × 10^−3^ (1.3 × 10^−3^)	<1.0 × 10^−9^
pLI762-Una	pWBG749e	OTUNa	5.7 × 10^−3^ (4.2 × 10^−3^)	<1.0 × 10^−9^
pLI50	pWBG749e-F7K	none	2.6 × 10^−4^ (2.9 × 10^−4^)	<1.0 × 10^−9^
pLI762–49	pWBG749e-F7K	OT49	1.2 × 10^−4^ (5.9 × 10^−5^)	2.6 × 10^−6^ (2.6 × 10^−6^)
pLI762–45	pWBG749e-F7K	OT45	1.4 × 10^−4^ (9.1 × 10^−5^)	1.3 × 10^−4^ (1.3 × 10^−4^)
pLI762-Una	pWBG749e-F7K	OTUNa	1.5 × 10^−4^ (1.5 × 10^−4^)	3.5 × 10^−5^ (4.4 × 10^−5^)

*Per-donor transfer frequencies are the average of three independent experiments (±standard deviation).

## DISCUSSION

In this work, we located SmpO-binding sites within the *oriT* subtypes present on five pWBG749-family plasmids. Each *oriT* contained two SmpO-binding sites named here *ossA* and *ossB*. The *ossB* sites were located within the right arm of the IR2 repeat sequence in each *oriT*, consistent with our previous proposal that the differences in mobilization specificities between pWBG749-family members were related to differences in IR2 repeat sequences (7). Alignments of pWBG749-family conjugation genes suggested some family members had switched their specificity between OT45 and OT49 *oriT* types during their evolutionary history. We introduced N-terminal substitutions in the SmpO_49_ and SmpO_45_ genes in an attempt to change their respective mobilization specificities. A single amino-acid change in either protein switched their specificities between OT49 and OT45 *oriT*s. A substituted SmpO_49-F7K_ variant bound the OT45 *oriT* in SPR and EMSA assays as efficiently as the SmpO_45_ protein. When this F7K substitution was introduced into the *smpO*_49_ gene on pWBG749e, mobilization preference was switched to the OT45 *oriT*, but the conjugation frequency of pWBG749e was only modestly reduced. Therefore, it seems the F7K substitution may be a viable evolutionary intermediate during the evolutionary switch in DNA-binding specificity between the OT49 and OT45 *oriT*s.

RHH DNA-binding proteins decode DNA sequences using an anti-parallel β-sheet encoded by the N-termini of each protein chain in the RHH domain (38). Several RHH proteins form tetramers and bind cooperatively to two or more binding sites (23,38–42). The TraM protein for instance forms tetramers that bind cooperatively to four 5′-GANTC-3′ motifs (40). In this work, EMSAs with the OT45 *oriT* revealed several complexes formed with increasing concentrations of SmpO_45_. SmpO proteins formed tetramers and hexamers in solution, so it is possible multiple SmpO proteins together bind *ossA* and *ossB* sites and form higher-order complexes. *In vivo* SmpO proteins may cooperatively bind *ossA* and *ossB*, perhaps together with the relaxase SmpP. This model could explain the relatively weak binding of *ossB* sites observed in some of the SPR assays, as *ossA* and *ossB* binding events were assayed in isolation from each other. Single nucleotide mutagenesis across three distinct *ossA* sites revealed SmpO proteins bound a ∼6–10 bp region centred around a conserved 5′-GNNNNC-3′motif. The importance of the central bases in this motif, which vary between *oriT* subtypes, implicates them as determinants of SmpO binding specificity. The distance between the centres of the *ossA* and *ossB* 5′-GNNNNC-3′ motifs ranged between 65 and 67 bp, suggesting spacing was also important, perhaps for higher order SmpO–*oriT* complex formation. The OTUNA and OTSep *ossA* sequences (and the OT45 *ossA* site on pWBG762) are present on the opposite DNA strand relative to the *ossB* site. Plasmids carrying SmpO_UNa_ or SmpO_45_ can each mobilize plasmids carrying either OTUNa or OT45 *oriT*, so the orientation of the *ossA* site relative to the *ossB* is clearly not critical. This is consistent with the dyad symmetry of the 5′-GNNNNC-3′ motif and the two-fold rotational symmetry of RHH domains.

In our earlier comparisons of the pWBG749-family *oriT* sequences we separated *oriT* sequences into subtypes based on global sequence alignments of the *oriT* sequences, which separated OT45 and OTUNa sites into distinct but closely related clusters. It is clear from subsequent work (4,17) and experiments here that the OT45 and OTUNa *oriT* are functionally interchangeable with respect to mobilization, despite being present on relatively distantly related members of the pWBG749 family. SPR assays suggest that despite this overlap in mobilization specificity, each of the SmpOs encoded by pWBG745 and pC02 still have a higher affinity for their cognate *oriT*s, as only SmpO_UNa_ produced weak SPR responses with the non-cognate OT45 *ossA* and *ossB* sites.

There were several discrepancies between our *in vitro* EMSA and SPR DNA-binding data and *in vivo* mobiliszation data. For instance, the SmpO_45-K7F_ variant was unable to bind OT49 *oriT* in SPR experiments (unpublished data) but the *smpO*_45-K7F_ allele enabled mobilization via the OT49 *oriT*. The *smpO*_45_ allele enabled mobilizations of plasmids carrying the OTUNa *oriT* yet we did not observe binding to the OTUNa *oriT* using SPR. Furthermore, the SmpO_49-F7K_ protein was able to bind the OT45 *oriT* but not the *ossA*_49_ or *ossB*_49_ in SPR experiments (Supplementary Table S4), yet the pWBG749e-F7K was able to conjugatively transfer via the OT49 *oriT* and mobilize a plasmid carrying the OT49 *oriT*, albeit at reduced rates. Some of the negative results for SPR experiments may result from incomplete optimization of conditions, relative positioning of the binding site on the oligonucleotide or perhaps the absence of required secondary DNA structures normally present *in vivo*. An alternative explanation is that the levels of *smpO* gene expression *in vivo* may be high enough to compensate for low-affinity binding by some of the SmpO proteins. Several RAFs including TraM negatively regulate their own expression by binding their own promoter. A negative autoregulation model for *smpO* gene expression would fit with some of the discrepancies observed. For instance, if SmpO_49_ represses transcription of *smpO*_49_ by binding the pWBG749 *oriT* region, then the weaker binding of SmpO_49-F7K_ to the *oriT* would result in SmpO_49-F7K_ overexpression, tentatively explaining how pWBG749e-F7K retains the ability to transfer from and mobilize plasmids carrying an OT49 *oriT*.

Conjugative plasmids with mobilization specificity for OT49 and OT45/OTUNa *oriT* both appear in two distinct branches of the pWBG749-family tree (Figure 5A), suggesting convergent evolution has occurred in at least one branch. It is possible that homologous recombination or relaxase-mediated recombination between two conjugative plasmids, or a conjugative plasmid and a mobilizable plasmid with an *oriT* mimic, may have led to the replacement of *oriT* sequences on conjugative-plasmids. Indeed, pWBG731 carries remnants of an ancestral recombination with a pC02-like plasmid (although outside the *oriT–smpO* region), illustrating that recombination between divergent pWBG749-family plasmids occurs. Moreover, the conjugative plasmid pC02 carries an additional OT49 *oriT* mimic outside its conjugation-gene cluster, so intramolecular recombination between the OT49 and OTUNa *oriTs* on pC02 could conceivably produce a plasmid resembling pM17027 (4,17). There is no compelling evidence however that the *smpO* sequences have been replaced through recombination on conjugative plasmids. While the *smpO* gene sequences are less conserved than surrounding conjugation genes on closely related plasmids with distinct *oriT* specificities, they are even less similar to *smpO* genes present on distantly related plasmids with the same *oriT* specificity (Figure 5B). Overall, these observations seem to best support a model in which *oriT* sequences have been replaced on conjugative plasmids through recombination while SmpO specificity has evolved through gain-of-function mutations.

We suspect the evolutionary partitioning of conjugation genes and beneficial gene cargo onto separate plasmids in *S. aureus* reflects a molecular symbiosis between conjugative plasmids and plasmids they mobilize. Staphylococcal plasmids are smaller than those found in many other bacteria. Roughly half of documented plasmids are <10 kb and the other half are mostly <40 kb (6). This strongly suggests some selective pressure restricts plasmid size in *S. aureus*. Conjugation-gene clusters, together with plasmid housekeeping loci such as replication and partitioning genes, typically span 27 kb alone, so it is not surprising that most identified pWBG749-family plasmids lack additional genetic cargo such as antimicrobial-resistance genes (6,7,10,43). Antimicrobial-resistance genes instead mostly appear as single resistance genes on small rolling-circle-replication plasmids or clustered together on 20–40 kb non-conjugative plasmids (1,3). For these larger plasmids (>10 kb), 85% carry a pWBG749-family *oriT* mimic, of which OT49 and OT45 are the dominant subtypes (44% and 47%, respectively) (7). Antimicrobial-resistance plasmids, without needing to dedicate large regions of DNA coding potential for conjugation functions, have space to collect suites of virulence and resistance genes while maintaining an ability to be mobilized horizontally by a variety of conjugative plasmids. Importantly, conjugative plasmids likely also benefit from this arrangement by sharing in the phenotypic advantages the mobilized plasmids endow when they transfer together to a new host. We suspect the apparent switching of *oriT* sequences on pWBG749-family plasmids may reflect instances where selection pressure exists for conjugative mobilization but the antimicrobial-resistance plasmid carries an incompatible *oriT* subtype. We demonstrated here that a single-codon change enables a conjugative plasmid to switch its *oriT* specificity to another *oriT* subtype while retaining an ability to self-transfer, thus providing a simple evolutionary pathway to enable mobilization of a plasmid carrying an otherwise incompatible *oriT* sequence.

The evolutionary pathways leading to changes in protein–DNA (and protein–protein) binding specificity are hotly debated (12,44–50). Evolution of a new DNA-binding specificity can be theoretically problematic depending on the evolutionary steps envisaged, which may in some scenarios involve the evolution of non-functional or redundant intermediary alleles. An alternative model that avoids non-functional intermediates is the evolution of a ‘promiscuous intermediate’ with broadened specificity for both the old DNA site and the new. pWBG749e-F7K mobilized plasmids carrying OT45/OTUNa and OT49 sites and while its mobilization preference favoured OT45, it remained capable of self-transfer from its own OT49 *oriT* albeit with reduced efficiency. While pWBG731 and pC02 plasmids also carry *smpO* alleles with a lysine at position 7, they do not mobilize plasmids carrying OT49 *oriT*s, suggesting SmpO_45_ and SmpO_UNa_ carry additional amino acid residues that prevent OT49 *oriT* binding. This raises the question if such promiscuous mobilizing plasmids persist in nature. Clearly, we have not tested all members of the pWBG749-family for their capacity to mobilize distinct *oriT* subtypes, so promiscuous variants may indeed exist.

pWBG749-family plasmids have clearly played an important role in the movement of antimicrobial-resistance plasmids in staphylococcal species, as evidenced by the widespread prevalence of *oriT* mimics carried by them. The pWBG749 family have diverged in *oriT* specificity during evolution but non-conjugative plasmids have kept pace by acquiring mimics corresponding to each subtype. In this work, we present evidence that pWBG749-family plasmids have converged on the same *oriT* specificities in distinct branches of the pWBG749-family tree. We present a feasible evolutionary pathway enabling such *oriT* specificity changes, in which a single amino-acid change can enable recognition of a previously incompatible site. Broadly, these observations suggest dynamic interplay between conjugative and mobilizable plasmids and further highlights the important role of horizontal transfer via conjugative mobilization.

## Supplementary Material

gkab303_Supplemental_Files
