# Supplementary material for: Evolving origin-of-transfer sequences on staphylococcal conjugative and mobilizable plasmids—who’s mimicking whom?
Source: Nucleic Acids Res. 2021 May 3;49(9):5177–88. doi: 10.1093/nar/gkab303 (PMC8136818; doi:10.1093/nar/gkab303)
Supplement: gkab303_Supplemental_Files [file gkab303_supplemental_files.zip › Supplementary_Information_22-3-2021_final.pdf]

Supporting Information

Table S1. Bacterial strains and plasmids

Strain/plasmid	Description	Reference
Staphylococcus aureus		
RN4220	Restriction-deficient derivate of NCTC8325-4 (RN450)	(1)
WBG4515	Str^R/Nov^R derivative of NCTC8325-4	(2)
WBG541	Fus^R/Rif^R derivative of NCTC8325-4	(2)
K102N	CC45, ST508 CA-MSSA carrying pWBG762 (Cad^R, Pen^R, Bla^+)	(3,4)
WBG10526	WBG541 carrying pWBG749e (pWBG749::Tn*551*) (Ery^R)	(5)
WBG8101	Original pWBG731 host	(6)
WBG8185	pWBG731 exconjugant following transfer from WBG8101	(6)
WBG10514	pWBG731 in WBG4515	This study
Escherichia coli		
EPI300	F^- *mcrA* Δ(*mcrCB-hsdSMR-mrr*) (Str^R) Φ80d*lacZ*ΔM15 Δ*lacX74 rec*A1 *end*A1 *araD*139 Δ(*ara*, *leu*)7697*galU galK* λ^- *rpsL nupG trfA ton*A *dhfr*	Epicentre
**Plasmids**		
pLI50	*S. aureus* / *E. coli* shuttle vector. Cm^R	(7)
pIMAY-Z	Allelic exchange vector for two-step gene replacement in staphylococci. Carries constitutively expressed *lacZ* gene and Cm^R	(8)
pWBG749e-F7K	pWBG749e with an introduced *smpO* allele encoding an F7K substitution, made using pIMAY-Z	
pLI749a	pLI50 carrying the *oriT* region of pWBG749	(9)
pLI762-49	pLI50 carrying the OT49-group *oriT* of pWBG762	(9)
pLI762-45	pLI50 carrying the OT45-group *oriT* of pWBG762	(9)
pLI762-UNa	pLI50 carrying the OTUNa-group *oriT* of pWBG762	(9)
pKY5T	pLI50 carrying *oriT* from pWBG745	(9)
pKY5TO	pLI50 carrying *oriT-smpO* region from pWBG745	(9)
pLI749a	pLI50 carrying the *oriT* region of pWBG749	(9)
pKY9TO	pLI50 carrying the *oriT-smpO* region of pWBG749 amplified using oligonucleotides 1 and 2	This study
pMA-T_S9	pMA-T carrying $SmpO_{49}$ mutagenesis cassette [MfeI-HindIII-OT45*oriT*-EcoRI-EcoRV-ClaI-$smpO_{49\text{-}3'}$-NarI], double-stranded fragment 63	This study
pLIOT5S9	pLI50 carrying pMA-T $SmpO_{49}$ mutagenesis cassette amplified using oligonucleotides 3 and 4 [HindIII-OT45oriT-EcoRI-EcoRV-ClaI-$smpO_{49\text{-}3'}$]	This study
pLIOT5S9M	pLIOT5S9 carrying annealed pairs of oligonucleotides (numbers 5-48) encoding the first 19 aa of $SmpO_{49}$ with a mixture of aa substitutions to match those aa present in the same positions on $SmpO_{45}$. A full list of plasmids is presented in Table S3.	This study
pLIS5	pLI50 carrying synthesized DNA (double-stranded oligonucleotide 64) containing the $SmpO_{45}$ mutagenesis cassette [HindIII-ClaI-$smpO_{45\text{-}3'}$] cloned as an EcoRI/ClaI fragment	This study
pLIOT9S5	pLIS5 carrying *oriT* from pWBG749 amplified using oligonucleotides 49 and 50 [HindIII-OT49*oriT*-EcoRI-ClaI-$smpO_{45\text{-}3'}$]	This study
pLIOT9S5-N-WT45	pLIOT9S5 carrying smpO_5′ from pWBG745, oligonucleotides 51 and 52	This study
pLIOT9S5-N-WT49	pLIOT9S5 carrying smpO_5′ from pWBG749, oligonucleotides 53 and 54	This study
pLIOT5S5M-K7K	pLIOT9S5 carrying smpO_5′ oligonucleotides 55 and 56 (carrying $SmpO_{45}$ mutation K7F)	This study
pETM-11	Protein expression vector for N-terminal hexahistidine fusions.	(10)
pETM-11_O49N	pETM-11 carrying *smpO* from pWBG749, amplified using oligonucleotides 57 and 58, cloned into NcoI/BamHI sites of pETM-11	This study
pETM-11_O45N	pETM-11 carrying *smpO* from pWBG745, amplified using oligonucleotides 58 and 59, cloned into NcoI/BamHI sites of pETM-11	This study
pETM-11_OUNA	pETM-11 carrying *smpO* from *S. aureus* W24216, cloned geneblock DNA $SmpO_{UNa}$_Ntag_XbaI_BamHI (double-stranded oligonucleotide 65) into XbaI/BamHI sites of pETM-11	This study
pETM-11_OSepN	pETM-11 carrying *smpO* from *S. aureus* VCU120, cloned geneblock DNA $SmpO_{Sep}$_Ntag_XbaI_BamHI (double-stranded oligonucleotide 66), and cloned into XbaI/BamHI sites of pETM-11	
pETM-11_O408N	pETM-11 carrying *smpO* from *S. aureus* M0408, cloned gblock DNA $SmpO_{408}$_Ntag_XbaI_BamHI (double-stranded oligonucleotide 67), at XbaI/BamHI sites of pETM-11	

pETM-11_O49-F7K_N	pETM-11 carrying *smpO* from pLIOT5S9M-F7K, amplified using oligonucleotides 60 and 61, cloned into NcoI/BamHI sites of pETM-11	This study
pETM-11_O45-K7F_N	pETM-11 carrying *smpO* from pLIOT9S5M-K7F, amplified using oligonucleotides 61 and 62, cloned into NcoI/BamHI sites of pETM-11	This study

Table S2. Oligonucleotides used for cloning

Number	Name	Sequence (5′ → 3′)
1	749_5'maximum_EcoRI	ATAAGAATTCATATCAAGCAACAAACAAAATAAGCTA
2	KYE_SmpO_BamH1_Rev	ATATGGATCCCATATCACCTACTCATTGAT
3	Kye_SmpO_product_Fwd	ATATCTAGATGCCAAAGATTGTAACAGTATCT
4	Kye_SmpO_product_Rev	ATATGGATCCCCTTTCTCTATATGATTGCT
5	WT_smp45_N-terminus_R	TCTCTTTATAGTTTCTAACATCAACGCTTCTTATTTTAATATCTCTTGTTTCCAT
6	WT_smp45_N-terminus_F	AATTATGGAAACAAGAGATATTAAAATAAGAAGCGTTGATGTAGAAACTATAAAGAGA
7	AC_smpO$_{49}$_4-7-12-14_O$_{45}$-F	AATTATGCCAACAAGAGATATTAAAATTAGGAACGTTGATGTAGAAACATTGGAGAGA
8	AC_smpO$_{49}$_4-7-12-14_O$_{45}$-R	TCTCTCCAATGTTTCTACATCAACGTTCCTAATTTTAATATCTCTTGTTGGCAT
9	AD-SmpO$_{49}$_7-10-12-17_O$_{45}$-F	AATTATGCCAACAAAAGATATTAAAATTAGGAGCGTTGATGTAGATACATTGAAGAGA
10	AD-SmpO$_{49}$_7-10-12-17_O$_{45}$-R	TCTCTTCAATGTATCTACATCAACGCTCCTAATTTTAATATCTTTTGT TGGCAT
11	BJ-SmpO$_{49}$_2_7_10_F	AATTATGGAAACAAAAGATATTAAAATTAGGAGCGTTAATGTAGATACATTGGAGAGA
12	BJ-SmpO$_{49}$_2_7_10_R	TCTCTCCAATGTATCTACATTAACGCTCCTAATTTTAATATCTTTTGT TTCCAT
13	BL-SmpO$_{49}$_7-12_O$_{45}$-F	AATTATGCCAACAAAAGATATTAAAATTAGAAACGTTGATGTAGATACATTGGAGAGA
14	BL-SmpO$_{49}$_7-12_O$_{45}$-R	TCTCTCCAATGTATCTACATCAACGTTTCTAATTTTAATATCTTTTGTTGGCAT
15	BS_SmpO$_{49}$_2-7-12-17_O$_{45}$-F	AATTATGGAAACAAAAGATATTAAAATTAGGAACGTTGATGTAGATACATTGAAGAGA
16	BS_SmpO$_{49}$_2-7-12-17_O$_{45}$-R	TCTCTTCAATGTATCTACATCAACGTTCCTAATTTTAATATCTTTTGTTTCCAT
17	IM_smpO$_{49}$_7-12-16_O$_{45}$-F	AATTATGCCAACAAAAGATATTAAAATTAGGAACGTTGATGTAGATACAATAGAGAGA
18	IM_smpO$_{49}$_7-12-16_O$_{45}$-R	TCTCTCTATTGTATCTACATCAACGTTCCTAATTTTAATATCTTTTGTTGGCAT
19	SmpO$_{49}$-2-7-12_JRAD-F	AATTATGGAAACAAAAGATATTAAAATTAGGAACGTTGATGTAGATACATTGGAGAGA
20	SmpO$_{49}$-2-7-12_JRAD-R	TCTCTCCAATGTATCTACATCAACGTTCCTAATTTTAATATCTTTTGTTTCCAT
21	LJ-SmpO$_{49}$_2-7-17_O$_{45}$-F	AATTATGGAAACAAAAGATATTAAAATTAGGAACGTTAATGTAGATACATTGAAGAGA
22	LJ-SmpO$_{49}$_2-7-17_O$_{45}$-R	TCTCTTCAATGTATCTACATTAACGTTCCTAATTTTAATATCTTTTGTTTCCAT
23	KYE_SmpO$_{49}$_2-4-7-10_F	AATTATGGAAACAAGAGATATTAAAATTAGGAGCGTTAATGTAGATACATTGGAGAGA
24	KYE_SmpO$_{49}$_2-4-7-10_R	TCTCTCCAATGTATCTACATTAACGCTCCTAATTTTAATATCTCTTGTTTCCAT
25	RJ_SmpO$_{49}$_8-15_O45-F	AATTATGCCAACAAAAGATATTAAAATAAGAAACGTTAATGTAGATACATTGAAGAGA
26	RJ_SmpO$_{49}$_8-15_O45-R	TCTCTTCAATGTATCTACATTAACGTTTCTTATTTTAATATCTTTTGTTGGCAT
27	SmpO$_{49}$_7FK_F	AATTATGCCAACAAAAGATATTAAAATTAGGAACGTTAATGTAGATACATTGGAGAGA
28	SmpO$_{49}$_7FK_R	TCTCTCCAATGTATCTACATTAACGTTCCTAATTTTAATATCTTTTGTTGGCAT
29	WT49_SmpO$_{49}$_F	AATTATGCCAACAAAAGATATTTTTATTAGGAACGTTAATGTAGATACATTGGAGAGA
30	WT49_SmpO$_{49}$_R	TCTCTCCAATGTATCTACATTAACGTTCCTAATAAAAATATCTTTTGTTGGCAT
31	CG-smpO$_{49}$__4-14_O$_{45}$-F	AATTATGCCAACAAGAGATATTTTTATTAGGAACGTTAATGTAGAAACATTGGAGAGA
32	CG-smpO$_{49}$__4-14_O$_{45}$-R	TCTCTCCAATGTTTCTACATTAACGTTCCTAATAAAAATATCTCTTGTTGGCAT
33	EB_smpO$_{49}$_2-4-14-17_O$_{45}$-F	AATTATGGAAACAAGAGATATTTTTATTAGGAACGTTAATGTAGAAACATTGAAGAGA
34	EB_smpO$_{49}$_2-4-14-17_O$_{45}$-R	TCTCTTCAATGTTTCTACATTAACGTTCCTAATAAAAATATCTCTTGTTTCCAT
35	HE-SmpO$_{49}$_12-14-17_O$_{45}$-F	AATTATGCCAACAAAAGATATTTTTATTAGGAACGTTGATGTAGAAACATTGAAGAGA
36	HE-SmpO$_{49}$_12-14-17_O$_{45}$-R	TCTCTTCAATGTTTCTACATCAACGTTCCTAATAAAAATATCTTTTGTTGGCAT
37	KK_smpO$_{49}$_2-12-14_O$_{45}$-F	AATTATGGAAACAAAAGATATTTTTATTAGGAACGTTGATGTAGAAACATTGGAGAGA
38	KK_smpO$_{49}$_2-12-14_O$_{45}$-R	TCTCTCCAATGTTTCTACATCAACGTTCCTAATAAAAATATCTTTTGTTTCCAT
39	MK_SmpO$_{49}$_2-17_O$_{45}$-F	AATTATGGAAACAAAAGATATTTTTATTAGGAACGTTAATGTAGATACATTGAAGAGA
40	MK_SmpO$_{49}$_2-17_O$_{45}$-R	TCTCTTCAATGTATCTACATTAACGTTCCTAATAAAAATATCTTTTGTTTCCAT
41	SmpO$_{49}$_4-10-16_NRDO-O$_{45}$-F	AATTATGCCAACAAGAGATATTTTTATTAGGAGCGTTAATGTAGATACAATAGAGAGA
42	SmpO$_{49}$_4-10-16_NRDO-O$_{45}$-R	TCTCTCTATTGTATCTACATTAACGCTCCTAATAAAAATATCTCTTGTTGGCAT
43	RM-smpO$_{49}$_10-12-14-17_O$_{45}$-F	AATTATGCCAACAAAAGATATTTTTATTAGGAGCGTTGATGTAGAAACATTGAAGAGA
44	RM-smpO$_{49}$_10-12-14-17_O$_{45}$-R	TCTCTTCAATGTTTCTACATCAACGCTCCTAATAAAAATATCTTTTGTTGGCAT
45	SM-SmpO$_{49}$_4-12-17_O$_{45}$-F	AATTATGCCAACAAGAGATATTTTTATTAGGAACGTTGATGTAGATACATTGAAGAGA
46	SM-SmpO$_{49}$_4-12-17_O$_{45}$-R	TCTCTTCAATGTATCTACATCAACGTTCCTAATAAAAATATCTCTTGTTGGCAT
47	KYE_SmpO$_{49}$_12-14-16-17_F	AATTATGCCAACAAAAGATATTTTTATTAGGAACGTTGATGTAGAAACAATAAAGAGA
48	KYE_SmpO$_{49}$_12-14-16-17_R	TCTCTTTATTGTTTCTACATCAACGTTCCTAATAAAAATATCTTTTGTTGGCAT
49	OT49-fwd-HindIII-Karina	ATCAAGCAACAAACAAAATAAGCTAAAAAAC
50	OT49-rev-claI-EcoRI-Karina	TTCTATCGATAATATGAATTCTGATAACCTCCCATAAAAATTAGCT
51	SmpO$_{45}$_F	AATTATGGAAACAAGAGATATTAAAATAAGAAGCGTTGATGTAGAAACTATAAAGAGAAT
52	SmpO$_{45}$_R	CGATTCTCTTTATAGTTTCTACATCAACGCTTCTTATTTTAATATCTCTTGTTTCCAT
53	SmpO$_{49}$_F	AATTATGCCAACAAAAGATATTTTTATTAGGAACGTTAATGTAGATACATTGGAGAGAAT
54	SmpO$_{49}$_R	CGATTCTCTCCAATGTATCTACATTAACGTTCCTAATAAAAATATCTTTTGTTGGCAT
55	SmpO$_{45}$_K7F_F	AATTATGGAAACAAGAGATATTTTTATAAGAAGCGTTGATGTAGAAACTATAAAGAGAAT
56	SmpO$_{45}$_K7F_R	CGATTCTCTTTATAGTTTCTACATCAACGCTTCTTATAAAAATATCTCTTGTTTCCAT
57	SmpO$_{49}$_Ntag_F_5’NcoI	ATATACCATGGCAATGCCAACAAAAGATATTTTTAT
58	KYE_SmpO_BamH1_Rev	ATATGGATCCCATATCACCTACTCATTGAT
59	SmpO$_{45}$_Ntag_F_5’NcoI	ATATCCATGGAAACAAGAGATATTAAAATAAGAAGC
60	SmpO$_{49}$_F7K_fwd_NcoI	AATTCCATGGATGCCAACAAAAGATATTAAAATTAGG
61	SmpO$_{45}$K7F_O49F7K_rev_BamHI_EcoRI	ATATGAATTCGGATCCTTAATATTCTAAATCATTAATAGTG
62	SmpO$_{45}$_K7F_fwd_NcoI	AATTCCATGGATGGAAACAAGAGATATTTTTATAAG

Table S2 continued. Oligonucleotides used for cloning

Number	Name	Sequence
63	SmpO$_{49}$ mutagenesis cassette [MfeI-HindIII-OT45*oriT*-EcoRI-EcoRV-ClaI-smpO$_{49\text{-}3'}$-NarI]<br>double-stranded DNA fragment	AATTCAATTGAAGCTTTTGAATAAAAAATATCAAGCAACAAACAAAATAAGCTAAAAAAC<br>AAGTGTCACAAAAATAAGACAAATTCTACATGTTGTGTCACAAAACTGTGACAAACACAA<br>TATATAGTGTCACAAAACTGTGACACTATAGTTTATTGTGGGGTCACTTTAAATTCTTTA<br>AAACCCTTGAAATGGCTGGCTTTGCCAGCCACCCCATAAAATATGGGGTCATATTTCCCT<br>TATGCTCTTACGAAAATTTTATTTTTAAAAGATTTTAAGTATTTTTGATTAGGAGGTTAA<br>CAGAATTCATATGATATCGATAGATTAGCAAAAAAACAAAAAAACATATCAAGAAACGAT<br>TTTTTATTAGGTATAATAGAACAGGTGGATCCGTTAGAATGTTACCGAAGTTTGTATGCC<br>GAACAATCACACCAACAAGCACAAAACACAAAGGCTTTAAAAGAATTAGCGGATAAAATA<br>GACCGAGTTTATAACACTATTAATGATTTAGAATATTAATGAGGCGCCAATA
64	SmpO$_{45}$ mutagenesis cassette [HindIII-ClaI-smpO$_{45\text{-}3'}$]<br>double-stranded oligonucleotide	AATTCAATTGAAGCTTAGAGAATCGATAGAATGGCAAAGCAAAAAAACATATCAAGGAAC<br>GAATTTTTATTGGACATAATAGAACAGATTGATCCATTAGAATGTTACCGAAGTTTGTAT<br>GCCGAACAATCTCACCAACAAGCACAAAATACGAAGGCTTTAAAAGAATTAGCGGATAAA<br>ATAGACCGAGTTTATAACACTATTAATGATTTAGAATATTAATGAGGCGCCGGGCG
65	SmpO$_{UNa}$_Ntag_XbaI_BamHI<br>double-stranded oligonucleotide	CGGGAACTAGTAGCGCACGCTGTCTAGAAAGCATTTTGATTTAACTTTAAGAAGGAGATA<br>TACCATGCACCATCATCACCACCACGAAAACCTGTACTTCCAGGGTATGGCTACCAAAGA<br>CCTGAAAGTTCGTAACGTTGACTCTGGTCTGCTGGAACGTCTGAACGTTGTTGCTGCTGA<br>ACAGAAAATCTCTCGTAACCAGCTGATCATCAACCTGCTGGAATCTCTGGACCCGCTGGA<br>ATCTTACCAGAAACTGTACGCTGAACAGACCCACCAGCAGGCTCAGAACACCAAAGTTCT<br>GAAAGAAGTTTGCTCTAAACAGGACGAAATCCTGAACATCCTGAAATCTGTTGACTACTA<br>AGGATCCTAGTGTCGAGCATTCAACCCCG
66	SmpO$_{Sep}$_Ntag_XbaI_BamHI<br>double-stranded oligonucleotide	CGGGAACTAGTAGCGCACGCTGTCTAGAAAGCATTTTGATTTAACTTTAAGAAGGAGATA<br>TACCATGCACCATCATCACCACCACGAAAACCTGTACTTCCAGGGTATGAAGAAGACCAA<br>GACCACCATCACCGTTCGTGACGTTGACCCGGAAATCAAAGAAAACCTGAAACAGGAAGC<br>TAACAAGAAAGGTCTGACCTTCAACAAATACATGAACGAACTGTTCGAAATCAACGACCC<br>GAAAGAAGTTTACAAGAAACTGTACGAAGAAACCTCTCACCAGCAGGCTATCTCTATCAA<br>AGTTATGAAAGAAATGACCAAGAAACTGGACGACATCCACAAAATCATGAAACAGATCGA<br>AGAAGAATAAGGATCCTAGTGTCGAGCATTCAACCCCG
67	SmpO$_{408}$_Ntag_XbaI_BamHI<br>double-stranded oligonucleotide	CGGGAACTAGTAGCGCACGCTGTCTAGAAAGCATTTTGATTTAACTTTAAGAAGGAGATA<br>TACCATGCACCATCATCACCACCACGAAAACCTGTACTTCCAGGGTATGAACGACTCTAA<br>AACCCGTCTGCACATCCGTAACATCGACGTTAACCTGAAACAGCGTCACCAGGAAATCGC<br>TAAAGAGAAAGGTCTGAACTTCTCTGACTACATCATCAAAATCCTGGAAGAAAACGACCC<br>GCTGGAACAGTACCGTAAACTGTACGAAGAAACCTCTCACCAGCAGGCTCAGACCCTGAA<br>AGTTATGAACGAACTGACCGAGAAAGTTGACAAAGTTTACAACATCATCAAACGTATCGA<br>AGACGAATAAGGATCCTAGTGTCGAGCATTCAACCCCG
68	pWBG749_smpO-1	CCTCACTAAAGGGAACAAAAGCTGGGTACCGCCAACGAAAGAAGATTACCAG
69	pWBG749_smpO-2	CTCATTGATTTAATATTCTGCTAATCGATCAATTCTCTCC
70	pWBG749_smpO-3	GATCGATTAGCAGAATATTAAATCAATGAGTAGG
71	pWBG749_smpO-4	CGACTCACTATAGGGCGAATTGGAGCTCGCAACATGAACATGTATGTTATCTG

Table S3. Oligonucleotides used for electrophoretic mobility shift assays and surface plasmon resonance assays

Name		Sequence	Reference
EMSA oligonucleotides			
IRadapterRev_ossA_WT		TTGAATGCTCGACACTATTGTGGGGTCACTTTAAATTCTTTAAAAC	This study
IRadapterRev_ossB_WT		GTAGCGCACGCTGAAGGGAAATATGACCCCATATTTTATGGGGTGG	This study
Universal_IR800_FWD		/5IRD800/TACGTGGTTGAATGCTCGACACTA	This study
Universal_IR800__REV		/5IRD800/TACGGGAACTAGTAGCGCACGCTG	This study
SPR Oligonucleotides			
ReDCaT linker		Biotin-gcaggaggacgtagggtagg	(11)
ReDCaT linker complement		cctaccctacgtcctcctgc	(11)
OT49 *oriT* sequence array			
OT49_V6For_1	OT49.1	ATATAGTGTCACAAAAATGTGACACTAGGT	This study
OT49_V6Rev_1		ACCTAGTGTCACATTTTTGTGACACTATATcctaccctacgtcctcctgc	This study
OT49_V6For_2	OT49.2	ACAAAAATGTGACACTAGGTGTTTTTATGA	This study
OT49_V6Rev_2		TCATAAAAACACCTAGTGTCACATTTTTGTcctaccctacgtcctcctgc	This study
OT49_V6For_3	OT49.3	GACACTAGGTGTTTTTATGATATCACTATG	This study
OT49_V6Rev_3		CATAGTGATATCATAAAAACACCTAGTGTCcctaccctacgtcctcctgc	This study
OT49_V6For_4	OT49.4	GTTTTTATGATATCACTATGAAATGTCCTA	This study
OT49_V6Rev_4		TAGGACATTTCATAGTGATATCATAAAAACcctaccctacgtcctcctgc	This study
OT49_V6For_5	OT49.5	TATCACTATGAAATGTCCTAAAACCCTTGG	This study
OT49_V6Rev_5		CCAAGGGTTTTAGGACATTTCATAGTGATAcctaccctacgtcctcctgc	This study
OT49_V6For_6	OT49.6	AAATGTCCTAAAACCCTTGGAATGTCTGGC	This study
OT49_V6Rev_6		GCCAGACATTCCAAGGGTTTTAGGACATTTcctaccctacgtcctcctgc	This study
OT49_V6For_7	OT49.7	AAACCCTTGGAATGTCTGGCTTTGCCAGAC	This study
OT49_V6Rev_7		GTCTGGCAAAGCCAGACATTCCAAGGGTTTcctaccctacgtcctcctgc	This study
OT49_V6For_8	OT49.8	AATGTCTGGCTTTGCCAGACCTATCATTGT	This study
OT49_V6Rev_8		ACAATGATAGGTCTGGCAAAGCCAGACATTcctaccctacgtcctcctgc	This study
OT49_V6For_9	OT49.9	TTTGCCAGACCTATCATTGTCCGATGATAG	This study
OT49_V6Rev_9		CTATCATCGGACAATGATAGGTCTGGCAAAcctaccctacgtcctcctgc	This study
OT49_V6For_10	OT49.10	CTATCATTGTCCGATGATAGCAAATTCCCC	This study
OT49_V6Rev_10		GGGGAATTTGCTATCATCGGACAATGATAGcctaccctacgtcctcctgc	This study
OT49_V6For_11	OT49.11	CCGATGATAGCAAATTCCCCTTATGCTCTT	This study
OT49_V6Rev_11		AAGAGCATAAGGGGAATTTGCTATCATCGGcctaccctacgtcctcctgc	This study
OT49_V6For_12	OT49.12	GATGATAGCAAATTCCCCTTATGCTCTTAC	This study
OT49_V6Rev_12		GTAAGAGCATAAGGGGAATTTGCTATCATCcctaccctacgtcctcctgc	This study
OT45 *oriT* sequence array			
OT45For_1_SPR	OT45.1	ATATAGTGTCACAAAACTGTGACACTATAG	This study
OT45Rev_1_SPR		CTATAGTGTCACAGTTTTGTGACACTATATcctaccctacgtcctcctgc	This study
OT45For_2_SPR	OT45.2	ACAAAACTGTGACACTATAGTTTATTGTGG	This study
OT45Rev_2_SPR		CCACAATAAACTATAGTGTCACAGTTTTGTcctaccctacgtcctcctgc	This study
OT45For_3_SPR	OT45.3	GACACTATAGTTTATTGTGGGGTCACTTTA	This study
OT45Rev_3_SPR		TAAAGTGACCCCACAATAAACTATAGTGTCcctaccctacgtcctcctgc	This study
OT45For_4_SPR	OT45.4	TTTATTGTGGGGTCACTTTAAATTCTTTAA	This study
OT45Rev_4_SPR		TTAAAGAATTTAAAGTGACCCCACAATAAAcctaccctacgtcctcctgc	This study
OT45For_5_SPR	OT45.5	GGTCACTTTAAATTCTTTAAAACCCTTGAA	This study
OT45Rev_5_SPR		TTCAAGGGTTTTAAAGAATTTAAAGTGACCcctaccctacgtcctcctgc	This study
OT45For_6_SPR	OT45.6	AATTCTTTAAAACCCTTGAAATGGCTGGCT	This study
OT45Rev_6_SPR		AGCCAGCCATTTCAAGGGTTTTAAAGAATTcctaccctacgtcctcctgc	This study
OT45For_7_SPR	OT45.7	AACCCTTGAAATGGCTGGCTTTGCCAGCCA	This study
OT45Rev_7_SPR		TGGCTGGCAAAGCCAGCCATTTCAAGGGTTcctaccctacgtcctcctgc	This study
OT45For_8_SPR	OT45.8	ATGGCTGGCTTTGCCAGCCACCCCATAAAA	This study
OT45Rev_8_SPR		TTTTATGGGGTGGCTGGCAAAGCCAGCCATcctaccctacgtcctcctgc	This study
OT45For_9_SPR	OT45.9	TTGCCAGCCACCCCATAAAATATGGGGTCA	This study
OT45Rev_9_SPR		TGACCCCATATTTTATGGGGTGGCTGGCAAcctaccctacgtcctcctgc	This study
OT45For_10_SPR	OT45.10	CCCCATAAAATATGGGGTCATATTTCCCTT	This study
OT45Rev_10_SPR		AAGGGAAATATGACCCCATATTTTATGGGGcctaccctacgtcctcctgc	This study
OT45For_11_SPR	OT45.11	TATGGGGTCATATTTCCCTTATGCTCTTAC	This study
OT45Rev_11_SPR		GTAAGAGCATAAGGGAAATATGACCCCATAcctaccctacgtcctcctgc	This study

Table S3 continued. Oligonucleotides used for electrophoretic mobility shift assays and surface plasmon resonance assays

Name		Sequence	Reference
OTUNa *oriT* sequence array			
OTUNaFor_7	OTUNA.1	CAATATATTGTGTCACAAAAGTGTGACAAT	This study
OTUNaRev_7		ATTGTCACACTTTTGTGACACAATATATTGcctaccctacgtcctcctgc	This study
OTUNaFor_8	OTUNA.2	TGTCACAAAAGTGTGACAATTTAATTTTTG	This study
OTUNaRev_8		CAAAAATTAAATTGTCACACTTTTGTGACAcctaccctacgtcctcctgc	This study
OTUNaFor_9	OTUNA.3	GTGTGACAATTTAATTTTTGTGACCCCCAT	This study
OTUNaRev_9		ATGGGGGTCACAAAAATTAAATTGTCACACcctaccctacgtcctcctgc	This study
OTUNaFor_10	OTUNA.4	TTAATTTTTGTGACCCCCATAAGATAAATG	This study
OTUNaRev_10		CATTTATCTTATGGGGGTCACAAAAATTAAcctaccctacgtcctcctgc	This study
OTUNaFor_11	OTUNA.5	TGACCCCCATAAGATAAATGTTTAAAACCC	This study
OTUNaRev_11		GGGTTTTAAACATTTATCTTATGGGGGTCAcctaccctacgtcctcctgc	This study
OTUNaFor_12	OTUNA.6	AAGATAAATGTTTAAAACCCTTGAAATATC	This study
OTUNaRev_12		GATATTTCAAGGGTTTTAAACATTTATCTTcctaccctacgtcctcctgc	This study
OTUNaFor_13	OTUNA.7	TTTAAAACCCTTGAAATATCTGGCTTCGCC	This study
OTUNaRev_13		GGCGAAGCCAGATATTTCAAGGGTTTTAAAcctaccctacgtcctcctgc	This study
OTUNaFor_14	OTUNA.8	TTGAAATATCTGGCTTCGCCAGATTACCCA	This study
OTUNaRev_14		TGGGTAATCTGGCGAAGCCAGATATTTCAAcctaccctacgtcctcctgc	This study
OTUNaFor_15	OTUNA.9	TGGCTTCGCCAGATTACCCATTATAAAATG	This study
OTUNaRev_15		CATTTTATAATGGGTAATCTGGCGAAGCCAcctaccctacgtcctcctgc	This study
OTUNaFor_16	OTUNA.10	AGATTACCCATTATAAAATGGGTACATATT	This study
OTUNaRev_16		AATATGTACCCATTTTATAATGGGTAATCTcctaccctacgtcctcctgc	This study
OTUNaFor_17	OTUNA.11	TTATAAAATGGGTACATATTTCCCTTATGC	This study
OTUNaRev_17		GCATAAGGGAAATATGTACCCATTTTATAAcctaccctacgtcctcctgc	This study
OTUNaFor_18	OTUNA.12	GGGTACATATTTCCCTTATGCTCTTACAAA	This study
OTUNaRev_18		TTTGTAAGAGCATAAGGGAAATATGTACCCcctaccctacgtcctcctgc	This study
OT408 *oriT* sequence array			
OT408For_7	OT408.1	GTACAGACACAATATATAGGGGTACATTTT	This study
OT408Rev_7		AAAATGTACCCCTATATATTGTGTCTGTACcctaccctacgtcctcctgc	This study
OT408For_8	OT408.2	AATATATAGGGGTACATTTTGGGTACAAAA	This study
OT408Rev_8		TTTTGTACCCAAAATGTACCCCTATATATTcctaccctacgtcctcctgc	This study
OT408For_9	OT408.3	GGTACATTTTGGGTACAAAACGGTTTTATA	This study
OT408Rev_9		TATAAAACCGTTTTGTACCCAAAATGTACCcctaccctacgtcctcctgc	This study
OT408For_10	OT408.4	GGGTACAAAACGGTTTTATAGGCGCCTAAT	This study
OT408Rev_10		ATTAGGCGCCTATAAAACCGTTTTGTACCCcctaccctacgtcctcctgc	This study
OT408For_11	OT408.5	CGGTTTTATAGGCGCCTAATAAAAACCTCT	This study
OT408Rev_11		AGAGGTTTTTATTAGGCGCCTATAAAACCGcctaccctacgtcctcctgc	This study
OT408For_12	OT408.6	GGCGCCTAATAAAAACCTCTGTATCCCTTG	This study
OT408Rev_12		CAAGGGATACAGAGGTTTTTATTAGGCGCCcctaccctacgtcctcctgc	This study
OT408For_13	OT408.7	AAAAACCTCTGTATCCCTTGAAATGTCTGG	This study
OT408Rev_13		CCAGACATTTCAAGGGATACAGAGGTTTTTcctaccctacgtcctcctgc	This study
OT408For_14	OT408.8	GTATCCCTTGAAATGTCTGGCTTCGCCAGA	This study
OT408Rev_14		TCTGGCGAAGCCAGACATTTCAAGGGATACcctaccctacgtcctcctgc	This study
OT408For_15	OT408.9	AAATGTCTGGCTTCGCCAGACCCACCTTTT	This study
OT408Rev_15		AAAAGGTGGGTCTGGCGAAGCCAGACATTTcctaccctacgtcctcctgc	This study
OT408For_16	OT408.10	CTTCGCCAGACCCACCTTTTTAATAGGTGG	This study
OT408Rev_16		CCACCTATTAAAAAGGTGGGTCTGGCGAAGcctaccctacgtcctcctgc	This study
OT408For_17	OT408.11	CCCACCTTTTTAATAGGTGGCAAATTTCCC	This study
OT408Rev_17		GGGAAATTTGCCACCTATTAAAAAGGTGGGcctaccctacgtcctcctgc	This study
OT408For_18	OT408.12	TAATAGGTGGCAAATTTCCCTTATGCTCTT	This study
OT408Rev_18		AAGAGCATAAGGGAAATTTGCCACCTATTAcctaccctacgtcctcctgc	This study
ossA SPR oligonuclotides			
OT49 min	ossA$_{49}$	TTTATGATATCACTAT	This study
OT49 min		ATAGTGATATCATAAAcctaccctacgtcctcctgc	This study
OT45For_min_SPR	ossA$_{45}$	ATTGTGGGGTCACTTT	This study
OT45Rev_min_SPR		AAAGTGACCCCACAATcctaccctacgtcctcctgc	This study
OTUNA_min_SPR_F	ossA$_{UNA}$	GTGACCCCCATAAGAT	This study
OTUNA_min_SPR_R		ATCTTATGGGGGTCACcctaccctacgtcctcctgc	This study
OTSep_min_SPR_F	ossA$_{Sep}$	TTTGCATACATTTAAA	This study
OTSep_min_SPR_R		TTAAATGTATGCAAAcctaccctacgtcctcctgc	This study
OT408_min_SPR_F	ossA$_{408}$	TTATAGGCGCCTAATA	This study
OT408_min_SPR_R		TATTAGGCGCCTATAAcctaccctacgtcctcctgc	This study

Table S3 continued. Oligonucleotides used for electrophoretic mobility shift assays and surface plasmon resonance assays

Name		Sequence	Reference
*ossA*49 mutagenesis array			
OT49IR2inv_F_1	T-ossA$_{49}$-1	ATTATGATATCACTAT	This study
OT49IR2inv_R_1		ATAGTGATATCATAATcctaccctacgtcctcctgc	This study
OT49IR2inv_F_2	T- ossA$_{49}$-2	TATATGATATCACTAT	This study
OT49IR2inv_R_2		ATAGTGATATCATATAcctaccctacgtcctcctgc	This study
OT49IR2inv_F_3	T- ossA$_{49}$-3	TTAATGATATCACTAT	This study
OT49IR2inv_R_3		ATAGTGATATCATTAAcctaccctacgtcctcctgc	This study
OT49IR2inv_F_4	A- ossA$_{49}$-4	TTTTTGATATCACTAT	This study
OT49IR2inv_R_4		ATAGTGATATCAAAAAcctaccctacgtcctcctgc	This study
OT49IR2inv_F_5	T- ossA$_{49}$-5	TTTAAGATATCACTAT	This study
OT49IR2inv_R_5		ATAGTGATATCTTAAAcctaccctacgtcctcctgc	This study
OT49IR2inv_F_6	G- ossA$_{49}$-6	TTTATCATATCACTAT	This study
OT49IR2inv_R_6		ATAGTGATATGATAAAcctaccctacgtcctcctgc	This study
OT49IR2inv_F_7	A- ossA$_{49}$-7	TTTATGTTATCACTAT	This study
OT49IR2inv_R_7		ATAGTGATAACATAAAcctaccctacgtcctcctgc	This study
OT49IR2inv_F_8	T- ossA$_{49}$-8	TTTATGAAATCACTAT	This study
OT49IR2inv_R_8		ATAGTGATTTCATAAAcctaccctacgtcctcctgc	This study
OT49IR2inv_F_9	A- ossA$_{49}$ -9	TTTATGATTTCACTAT	This study
OT49IR2inv_R_9		ATAGTGAAATCATAAAcctaccctacgtcctcctgc	This study
OT49IR2inv_F_10	T- ossA$_{49}$-10	TTTATGATAACACTAT	This study
OT49IR2inv_R_10		ATAGTGTTATCATAAAcctaccctacgtcctcctgc	This study
OT49IR2inv_F_11	C- ossA$_{49}$-11	TTTATGATATGACTAT	This study
OT49IR2inv_R_11		ATAGTCATATCATAAAcctaccctacgtcctcctgc	This study
OT49IR2inv_F_12	A- ossA$_{49}$-12	TTTATGATATCTCTAT	This study
OT49IR2inv_R_12		ATAGAGATATCATAAAcctaccctacgtcctcctgc	This study
OT49IR2inv_F_13	C- ossA$_{49}$-13	TTTATGATATCAGTAT	This study
OT49IR2inv_R_13		ATACTGATATCATAAAcctaccctacgtcctcctgc	This study
OT49IR2inv_F_14	T- ossA$_{49}$-14	TTTATGATATCACAAT	This study
OT49IR2inv_R_14		ATTGTGATATCATAAAcctaccctacgtcctcctgc	This study
OT49IR2inv_F_15	A- ossA$_{49}$-15	TTTATGATATCACTT T	This study
OT49IR2inv_R_15		AAAGTGATATCATAAAcctaccctacgtcctcctgc	This study
OT49IR2inv_F_16	T- ossA$_{49}$-16	TTTATGATATCACTAA	This study
OT49IR2inv_R_16		TTAGTGATATCATAAAcctaccctacgtcctcctgc	This study
*ossA*45 mutagenesis array			
OT45_min_1F	A- ossA$_{45}$-1	TTTGTGGGGTCACTTT	This study
OT45_min_1R		AAAGTGACCCCACAAAcctaccctacgtcctcctgc	This study
OT45_min_2F	T- ossA$_{45}$-2	AATGTGGGGTCACTTT	This study
OT45_min_2R		AAAGTGACCCCACATTcctaccctacgtcctcctgc	This study
OT45_min_3F	T- ossA$_{45}$-3	ATAGTGGGGTCACTTT	This study
OT45_min_3R		AAAGTGACCCCACTATcctaccctacgtcctcctgc	This study
OT45_min_4F	G- ossA$_{45}$-4	ATTCTGGGGTCACTTT	This study
OT45_min_4R		AAAGTGACCCCAGAATcctaccctacgtcctcctgc	This study
OT45_min_5F	T- ossA$_{45}$-5	ATTGAGGGGTCACTTT	This study
OT45_min_5R		AAAGTGACCCCTCAATcctaccctacgtcctcctgc	This study
OT45_min_6F	G- ossA$_{45}$-6	ATTGTCGGGTCACTTT	This study
OT45_min_6R		AAAGTGACCCGACAATcctaccctacgtcctcctgc	This study
OT45_min_7F	G- ossA$_{45}$-7	ATTGTGCGGTCACTTT	This study
OT45_min_7R		AAAGTGACCGCACAATcctaccctacgtcctcctgc	This study
OT45_min_8F	G- ossA$_{45}$-8	ATTGTGGCGTCACTTT	This study
OT45_min_8R		AAAGTGACGCCACAATcctaccctacgtcctcctgc	This study
OT45_min_9F	G- ossA$_{45}$-9	ATTGTGGGCTCACTTT	This study
OT45_min_9R		AAAGTGAGCCCACAATcctaccctacgtcctcctgc	This study
OT45_min_10F	T- ossA$_{45}$-10	ATTGTGGGGACACTTT	This study
OT45_min_10R		AAAGTGTCCCCACAATcctaccctacgtcctcctgc	This study
OT45_min_11F	C- ossA$_{45}$-11	ATTGTGGGGTGACTTT	This study
OT45_min_11R		AAAGTCACCCCACAATcctaccctacgtcctcctgc	This study
OT45_min_12F	A- ossA$_{45}$-12	ATTGTGGGGTCTCTTT	This study
OT45_min_12R		AAAGAGACCCCACAATcctaccctacgtcctcctgc	This study
OT45_min_13F	C- ossA$_{45}$-13	ATTGTGGGGTCAGTTT	This study
OT45_min_13R		AAACTGACCCCACAATcctaccctacgtcctcctgc	This study
OT45_min_14F	T- ossA$_{45}$-14	ATTGTGGGGTCACATT	This study
OT45_min_14R		AATGTGACCCCACAATcctaccctacgtcctcctgc	This study

Table S3 continued. Oligonucleotides used for electrophoretic mobility shift assays and surface plasmon resonance assays

Name		Sequence	Reference
ossA$_{45}$ mutagenesis array			
OT45_min_15F	T- ossA$_{45}$-15	ATTGTGGGGTCACTAT	This study
OT45_min_15R		ATAGTGACCCCACAATcctaccctacgtcctcctgc	This study
OT45_min_16F	T- ossA$_{45}$-16	ATTGTGGGGTCACTTA	This study
OT45_min_16R		TAAGTGACCCCACAATcctaccctacgtcctcctgc	This study
ossA$_{408}$ mutagenesis array			
408min_mut_F1	T-ossA$_{408}$-1	ATATAGGCGCCTAATA	This study
408min_mut_R1		TATTAGGCGCCTATATcctaccctacgtcctcctgc	This study
408min_mut_F2	T-ossA$_{408}$-2	TAATAGGCGCCTAATA	This study
408min_mut_R2		TATTAGGCGCCTATTAcctaccctacgtcctcctgc	This study
408min_mut_F3	A-ossA$_{408}$-3	TTTTAGGCGCCTAATA	This study
408min_mut_R3		TATTAGGCGCCTAAAAcctaccctacgtcctcctgc	This study
408min_mut_F4	T-ossA$_{408}$-4	TTAAAGGCGCCTAATA	This study
408min_mut_R4		TATTAGGCGCCTTTAAcctaccctacgtcctcctgc	This study
408min_mut_F5	A-ossA$_{408}$-5	TTATTGGCGCCTAATA	This study
408min_mut_R5		TATTAGGCGCCAATAAcctaccctacgtcctcctgc	This study
408min_mut_F6	G-ossA$_{408}$-6	TTATACGCGCCTAATA	This study
408min_mut_R6		TATTAGGCGCGTATAAcctaccctacgtcctcctgc	This study
408min_mut_F7	G-ossA$_{408}$-7	TTATAGCCGCCTAATA	This study
408min_mut_R7		TATTAGGCGGCTATAAcctaccctacgtcctcctgc	This study
408min_mut_F8	C-ossA$_{408}$-8	TTATAGGGGCCTAATA	This study
408min_mut_R8		TATTAGGCCCCTATAAcctaccctacgtcctcctgc	This study
408min_mut_F9	G-ossA$_{408}$-9	TTATAGGCCCCTAATA	This study
408min_mut_R9		TATTAGGGGCCTATAAcctaccctacgtcctcctgc	This study
408min_mut_F10	C-ossA$_{408}$-10	TTATAGGCGGCTAATA	This study
408min_mut_R10		TATTAGCCGCCTATAAcctaccctacgtcctcctgc	This study
408min_mut_F11	C-ossA$_{408}$-11	TTATAGGCGCGTAATA	This study
408min_mut_R11		TATTTGGCGCCTATAAcctaccctacgtcctcctgc	This study
408min_mut_F12	T-ossA$_{408}$-12	TTATAGGCGCCAAATA	This study
408min_mut_R12		TATTTGGCGCCTATAAcctaccctacgtcctcctgc	This study
408min_mut_F13	A-ossA$_{408}$-13	TTATAGGCGCCTTATA	This study
408min_mut_R13		TATAAGGCGCCTATAAcctaccctacgtcctcctgc	This study
408min_mut_F14	A-ossA$_{408}$-14	TTATAGGCGCCTATTA	This study
408min_mut_R14		TAATAGGCGCCTATAAcctaccctacgtcctcctgc	This study
408min_mut_F15	T-ossA$_{408}$-15	TTATAGGCGCCTAAAA	This study
408min_mut_R15		TTTTAGGCGCCTATAAcctaccctacgtcctcctgc	This study
408min_mut_F16	A-ossA$_{408}$-16	TTATAGGCGCCTAATT	This study
408min_mut_R16		AATTAGGCGCCTATAAcctaccctacgtcctcctgc	This study

Table S4. SPR responses for each SmpO protein with each *ossA* and *ossB* site.

	$SmpO_{45}$	$SmpO_{49\text{-}F7K}$	$SmpO_{49}$	$SmpO_{UNa}$	$SmpO_{408}$	$SmpO_{Sep}$
$ossA_{45}$	37.5	31.3	0.1	7.1	10.7	2.9
$ossB_{45}$	21.6	26.1	0.2	1.4	0.9	0.5
$ossA_{49}$	0	0	5	0	0.1	1.2
$ossB_{49}$	0	0	0	0	0	0.6
$ossA_{UNa}$	1.6	3.5	0	12.4	2.9	0
$ossB_{UNa}$	0.8	0	0.1	0.2	0	1.5
$ossA_{408}$	0	0	0	0	52.8	0
$ossB_{408}$	0.4	0	0.2	0	34.2	0.1
$ossA_{Sep}$	0	0	0	0	0	18.8
$ossB_{Sep}$	0.3	0	0.1	0	0	16.5

%R_{max} responses averaged from two independent experiments, complete SPR data are presented Supporting Dataset S1

Table S5. SEC-MALS analyses of SmpO proteins

Parameters	BSA[a]		SmpO$_{49}$		SmpO$_{45}$		SmpO$_{408}$	
	**Dimer**	**Monomer**	**1**	**2**	**1**	**2**	**1**	**2**
**Theoretical MW (g/mol)**	133,400	66,700	10,227		10,158		10,572	
**Abs 0.1% (=1g/L)**	0.660	0.660	0.583		0.587		0.427	
**Retention volume (mL)**	12.99 ± 0.00	14.97 ± 0.00	13.34 ± 0.00	16.04 ± 0.00	13.36 ± 0.00	15.78 ± 0.00	13.41 ± 0.00	15.40 ± 0.00
**Mn (g/mol)**	133,715 ± 10,187	66,093 ± 343	58,120 ± 2,812	40,244 ± 1,923	64,508 ± 4,625	40,347 ± 2,787	60,820 ± 4,269	43,683 ± 3,042
**Mw (g/mol)**	**134,472 ± 10,289**	**66,584 ± 367**	**58,221 ± 2,840**	**40,372 ± 1,956**	**64,673 ± 4,592**	**40,638 ± 2,830**	**60,859 ± 4,287**	**43,911 ± 3,084**
**Mz (g/mol)**	135,139 ± 10,447	67,211 ± 291	58,317 ± 2,866	40,473 ± 1,981	64,852 ± 4.560	40,869 ± 2,867	60,899 ± 4,305	44,135 ± 3,129
**Mw/Mn**	1.006 ± 0.003	1.008 ± 0.002	1.002 ± 0.001	1.003 ± 0.001	1.003 ± 0.002	1.007 ± 0.002	1.001 ± 0.001	1.005 ± 0.001
**Mw/predicted Mw**	1.008	0.998	5.69	3.95	6.37	3.98	5.76	4.15
**Total Weight Fraction (%)**	20.97 ± 0.92	69.56 ± 1.62	15.78 ± 0.08	84.22 ± 0.08	3.40 ± 0.00	96.60 ± 0.00	6.66 ± 0.00	93.34 ± 0.00
**Input conc (mg/mL)**	0.950		2.651		2.8220		2.200	
**Calculated Conc (mg/mL)**	0.853 ± 0.036		2.763 ± 0.173		2.364 ± 0.297		1.903 ± 0.239	

**Parameters**	**Parameters**	SmpO$_{49\text{-}F7K}$		SmpO$_{45\text{-}K7F}$		SmpO$_{Sep}$	
		**1**	**2**	**1**	**2**	**1**	**2**
**Theoretical MW (g/mol)**		10,210		10,177		10,475	
**Abs 0.1% (=1g/L)**		0.584		0.586		0.564	
**Retention volume (mL)**		13.36 ± 0.00	15.77 ± 0.00	14,71 ± 0.02	16.07 ± 0.00	13.37 ± 0.00	15.54 ± 0.00
**Mn (g/mol)**		61,727 ± 4.348	40,949 ± 2,855	64,695 ± 4,701	39,136 ± 2,727	57,421 ± 4,011	40,847 ± 2,852
**Mw (g/mol)**		**61,851 ± 4,371**	**41,026 ± 2,877**	**65,187 ± 4,674**	**39,208 ± 2,750**	**57,560 ± 4,042**	**41,037 ± 2,887**
**Mz (g/mol)**		61,996 ± 4,398	41,098 ± 2,898	65,668 ± 4,654	39,274 ± 2,769	57,681 ± 4,069	41,189 ± 2,914
**Mw/Mn**		1.002 ± 0.001	1.002 ± 0.001	1.008 ± 0.001	1.002 ± 0.001	1.002 ± 0.002	1.005 ± 0.001
**Mw/predicted Mw**		6.06	4.02	6.40	3.85	5.49	3.92
**Total Weight Fraction (%)**		7.62 ± 0.00	92.38 ± 0.00	3.53 ± 0.00	96.47 ± 0.00	9.76 ± 0.00	90.24 ± 0.00
**Input conc (mg/mL)**		2.583		2.832		2.450	
**Calculated Conc (mg/mL)**		2.449 ± 0.308		2.288± 0.287		2.120 ± 0.266	

*1 and 2 refer to detected peak 1 and peak 2 in size-exclusion profiles. Mn, Mw and Mz respectively correspond to the number-, weight- and z-average molecular weight. Mw results are shown in bold. Mw/predicted Mw = oligomeric state of the proteins. Input concentration refers to concentration derived from 280 nm absorbance nanodrop reading and calculated concentration refers to concentration calculated by the SEC-MALS software. [a] BSA trimer data is not shown. Refer to text for more details.

Table S6. $SmpO_{49}$ β-sheet mutagenesis and pWBG749e-mediated conjugative mobilisation of an OT45-type *oriT*

Vector name	Oligonucleotides cloned	SmpO variant[a]	Transfer frequency[b] pWBG749e	pLI50 clone
pLI50	vector-only control	vector-only control	3.1×10^{-5} (± 2.4×10^{-5})	Not detected
pKY5TO	positive control	$SmpO_{45}$	1.7×10^{-5} (± 1.6×10^{-6})	8.0×10^{-6} (± 9.4×10^{-7})
pLIOT5S9M-N-WT45	7 & 8	$SmpO_{45}$(1-17)-$SmpO_{49}$(18-84)	9.8×10^{-6} (± 4.1×10^{-6})	1.7×10^{-6} (± 7.8×10^{-7})
pLIOT5S9M-4-7-12-14	9 & 10	$SmpO_{45}$(4,7,12,14)-$SmpO_{49}$(18-84)	3.4×10^{-5} (± 2.1×10^{-5})	1.3×10^{-5} (± 4.9×10^{-6})
pLIOT5S9M-7-10-12-17	11 & 12	$SmpO_{45}$7,10,12,17)-$SmpO_{49}$(18-84)	3.0×10^{-5} (± 3.5×10^{-5})	4.6×10^{-6} (± 5.4×10^{-6})
pLIOT5S9M-2-7-10	13 & 14	$SmpO_{45}$(2,7,10)-$SmpO_{49}$(18-84)	2.4×10^{-5} (± 1.6×10^{-2})	8.0×10^{-6} (± 5.2×10^{-6})
pLIOT5S9M-7-12	15 & 16	$SmpO_{45}$(7,12)-$SmpO_{49}$(18-84)	1.9×10^{-5} (± 1.2×10^{-5})	9.3×10^{-6} (± 5.5×10^{-6})
pLIOT5S9M-2-7-12-17	17 & 18	$SmpO_{45}$(2,7,12,17)-$SmpO_{49}$(18-84)	3.8×10^{-5} (± 6.9×10^{-6})	1.3×10^{-5} (± $3.2x 10^{-6}$)
pLIOT5S9-7-12-16	19 & 20	$SmpO_{45}$(7,12,16)-$SmpO_{49}$(18-84)	1.2×10^{-5} (± 5.9×10^{-6})	7.3×10^{-6} (± 3.7×10^{-6})
pLIOT5S9M-2-7-12	21 & 22	$SmpO_{45}$(2,7,12)-$SmpO_{49}$(18-84)	1.2×10^{-5} (± 1.1×10^{-5})	7.2×10^{-6} (± 5.7×10^{-6})
pLIOT5S9M-2-7-17	23 & 24	$SmpO_{45}$(2,7,17)-$SmpO_{49}$(18-84)	1.5×10^{-5} (± 9.9×10^{-6})	7.0×10^{-6} (± 5.7×10^{-6})
pLIOT5S9M-2-4-7-10	25 & 26	$SmpO_{45}$(2,4,7,10)-$SmpO_{49}$(18-84)	1.5×10^{-5} (± 7.1×10^{-6})	3.7×10^{-6} (± 1.7×10^{-6})
pLIOT5S9M-7-17	27 & 28	$SmpO_{45}$(7,17)-$SmpO_{49}$(18-84)	1.8×10^{-5} (± 1.2×10^{-5})	4.5×10^{-6} (± $3.0x 10^{-6}$)
pLIOT5S9M-F7K	29 & 30	$SmpO_{49\text{-}F7K}$	9.6×10^{-6} (± 7.4×10^{-6})	4.3×10^{-6} (± 4.9×10^{-6})
pLIOT5S9M-N-WT49	31 & 32	$SmpO_{49}$	1.7×10^{-5} (± 1.5×10^{-5})	Not detected
pLIOT5S9M-4-14	33 & 34	$SmpO_{45}$(4,14)-$SmpO_{49}$(18-84)	3.2×10^{-5} (± 2.3×10^{-5})	Not detected
pLIOT5S9M-2-4-14-17	35 & 36	$SmpO_{45}$(2,4,14,17)-$SmpO_{49}$(18-84)	3.1×10^{-5} (± 1.2×10^{-5})	Not detected
pLIOT5S9M-12-14-17	37 & 38	$SmpO_{45}$(12,14,17)-$SmpO_{49}$(18-84)	1.3×10^{-5} (± 7.3×10^{-6})	Not detected
pLIOT5S9M-2-12-14	39 & 40	$SmpO_{45}$(2,12,14)-$SmpO_{49}$(18-84)	1.5×10^{-5} (± 1.1×10^{-5})	Not detected
pLIOT5S9M-2-17	41 & 42	$SmpO_{45}$(2,17)-$SmpO_{49}$(18-84)	2.2×10^{-5} (± 1.5×10^{-5})	Not detected
pLIOT5S9M-4-10-16	43 & 44	$SmpO_{45}$(4,10,16)-$SmpO_{49}$(18-84)	3.1×10^{-5} (± 9.1×10^{-6})	Not detected
pLIOT5S9M-10-12-14-17	45 & 46	$SmpO_{45}$(10,12,14,17)-$SmpO_{49}$(18-84)	1.5×10^{-5} (± 8.0×10^{-6})	Not detected
pLIOT5S9M-4-12-17	47 & 48	$SmpO_{45}$(4,12,17)-$SmpO_{49}$(18-84)	1.8×10^{-5} (± 6.6×10^{-6})	Not detected
pLIOT5S9M-12-14-16-17	49 & 50	$SmpO_{45}$(12,14,16,17)-$SmpO_{49}$(18-84)	1.1×10^{-5} (± 6.9×10^{-6})	Not detected

[a] Numbers in brackets correspond to regions or residues mutated to match those present in the named SmpO protein.

[b]Per-donor transfer frequencies are the average of three independent experiments (±standard deviation)

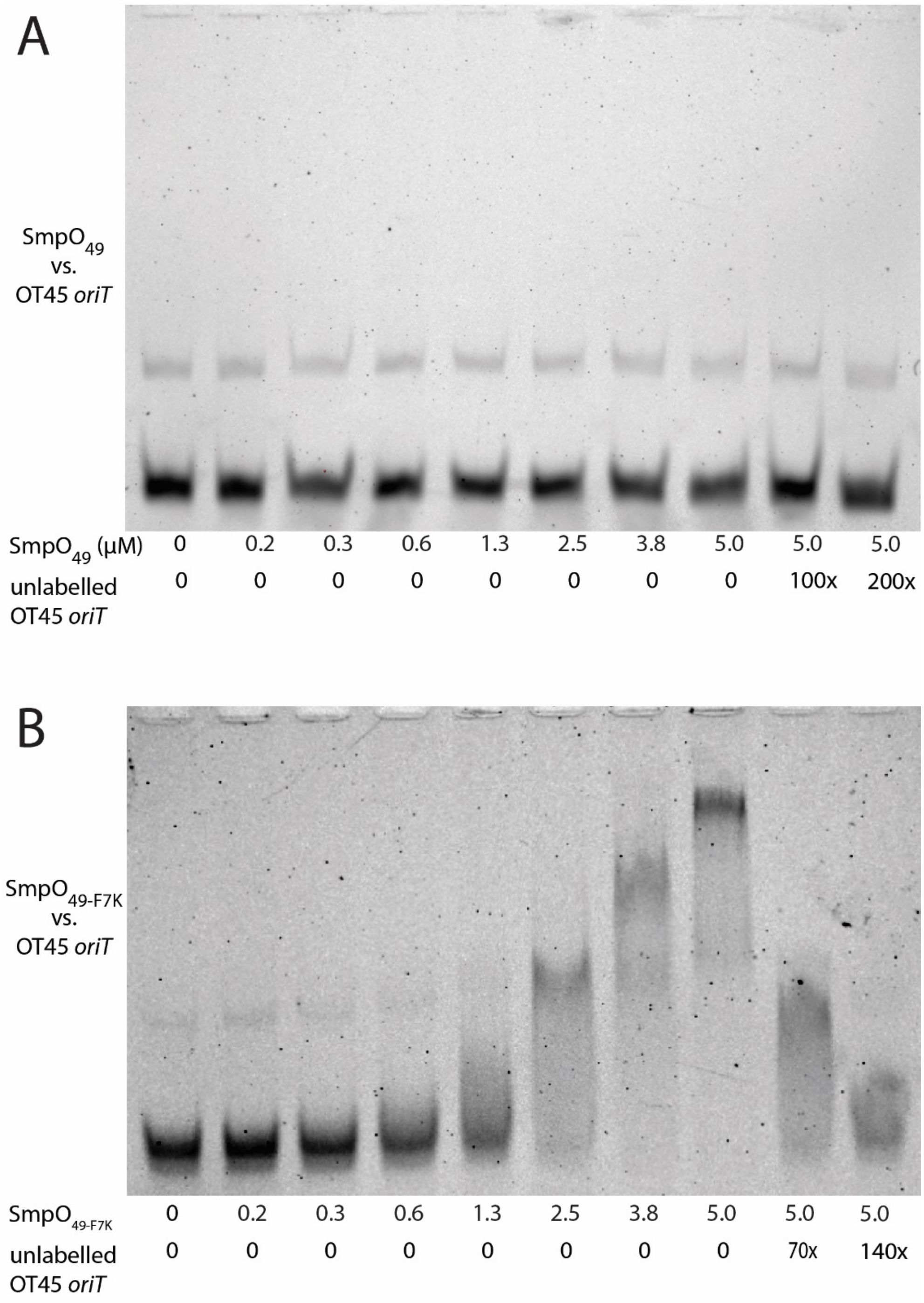

Figure S1. EMSA of SmpO49 and SmpO49-F7K with the OT45 *oriT*. Purified $SmpO_{49}$ and $SmpO_{49\text{-}F7K}$ were used in EMSA assays with IRDye800-tagged DNA comprising a 134 bp region encompassing the IR1-IR3 for *oriT* sequence OT45. (A) Increasing concentrations of purified $SmpO_{49}$ were added to 5 nM IRDye800-labelled OT45. (B) Increasing concentrations of purified $SmpO_{49\text{-}F7K}$ were added to 5 nM concentration of IRDye800-labelled OT45. The final two lanes show competing unlabelled OT45 DNA inhibits shifting of the labelled DNA Secondary labelled DNA with an apparent higher molecular weight were visible for labelled OT45 and were shifted in a similar manner to the main PCR products

References

1. Kreiswirth, B.N., Lofdahl, S., Betley, M.J., O'Reilly, M., Schlievert, P.M., Bergdoll, M.S. and Novick, R.P. (1983) The toxic shock syndrome exotoxin structural gene is not detectably transmitted by a prophage. *Nature*, **305**, 709-712.
2. Townsend, D.E., Grubb, W.B. and Ashdown, N. (1983) Gentamicin resistance in methicillin-resistant *Staphylococcus aureus*. *Pathology*, **15**, 169-174.
3. O'Brien, F.G., Coombs, G.W., Pearman, J.W., Gracey, M., Moss, F., Christiansen, K.J. and Grubb, W.B. (2009) Population dynamics of methicillin-susceptible and -resistant *Staphylococcus aureus* in remote communities. *The Journal of antimicrobial chemotherapy*, **64**, 684-693.
4. Shearer, J.E., Wireman, J., Hostetler, J., Forberger, H., Borman, J., Gill, J., Sanchez, S., Mankin, A., Lamarre, J., Lindsay, J.A. *et al.* (2011) Major families of multiresistant plasmids from geographically and epidemiologically diverse staphylococci. *G3 (Bethesda)*, **1**, 581-591.
5. O'Brien, F.G., Ramsay, J.P., Monecke, S., Coombs, G.W., Robinson, O.J., Htet, Z., Alshaikh, F.A.M. and Grubb, W.B. (2015) *Staphylococcus aureus* plasmids without mobilization genes are mobilized by a novel conjugative plasmid from community isolates. *The Journal of antimicrobial chemotherapy*, **70**, 649-652.
6. Slattery, J.T., Udo, E.E., Pearman, J.W., Riley, T. and Grubb, W.B. (1995), *Australian Society for Microbiology Annual Scientific Meeting (in Canberra)*, Vol. 16, pp. 24-29.
7. Lee, C.Y., Buranen, S.L. and Ye, Z.H. (1991) Construction of single-copy integration vectors for *Staphylococcus aureus*. *Gene*, **103**, 101-105.
8. Monk, I.R., Tree, J.J., Howden, B.P., Stinear, T.P. and Foster, T.J. (2015) Complete Bypass of Restriction Systems for Major *Staphylococcus aureus* Lineages. *mBio*, **6**, e00308-00315.
9. O'Brien, F.G., Yui Eto, K., Murphy, R.J., Fairhurst, H.M., Coombs, G.W., Grubb, W.B. and Ramsay, J.P. (2015) Origin-of-transfer sequences facilitate mobilisation of non-conjugative antimicrobial-resistance plasmids in *Staphylococcus aureus*. *Nucleic Acids Res*, **43**, 7971-7983.
10. Dummler, A., Lawrence, A.M. and de Marco, A. (2005) Simplified screening for the detection of soluble fusion constructs expressed in E. coli using a modular set of vectors. *Microb Cell Fact*, **4**, 34.
11. Stevenson, C.E., Assaad, A., Chandra, G., Le, T.B., Greive, S.J., Bibb, M.J. and Lawson, D.M. (2013) Investigation of DNA sequence recognition by a streptomycete MarR family transcriptional regulator through surface plasmon resonance and X-ray crystallography. *Nucleic Acids Res*, **41**, 7009-7022.
